# Oriented electron transmission in polyoxometalate-metalloporphyrin organic framework for highly selective electroreduction of CO_2_

**DOI:** 10.1038/s41467-018-06938-z

**Published:** 2018-10-26

**Authors:** Yi-Rong Wang, Qing Huang, Chun-Ting He, Yifa Chen, Jiang Liu, Feng-Cui Shen, Ya-Qian Lan

**Affiliations:** 1 Jiangsu Collaborative Innovation Centre of Biomedical Functional Materials, Jiangsu Key Laboratory of New Power Batteries, School of Chemistry and Materials Science, Nanjing Normal University, 210023 Nanjing, China; 2 MOE Key Laboratory of Functional Small Organic Molecule College of Chemistry and Chemical Engineering, Jiangxi Normal University, 330022 Nanchang, China

## Abstract

The design of highly stable, selective and efficient electrocatalysts for CO_2_ reduction reaction is desirable while largely unmet. In this work, a series of precisely designed polyoxometalate-metalloporphyrin organic frameworks are developed. Noted that the integration of {ε-PMo_8_^V^Mo_4_^VI^O_40_Zn_4_} cluster and metalloporphyrin endows these polyoxometalate-metalloporphyrin organic frameworks greatly advantages in terms of electron collecting and donating, electron migration and electrocatalytic active component in the CO_2_ reduction reaction. Thus-obtained catalysts finally present excellent performances and the mechanisms of catalysis processes are discussed and revealed by density functional theory calculations. Most importantly, Co-PMOF exhibits remarkable faradaic efficiency ( > 94%) over a wide potential range (−0.8 to −1.0 V). Its best faradaic efficiency can reach up to 99% (highest in reported metal-organic frameworks) and it exhibits a high turnover frequency of 1656 h^−1^ and excellent catalysis stability ( > 36 h).

## Abstract (web summary)

While CO2 reduction provides a way to remove carbon from the atmosphere, it is challenging to design effective, selective materials for this process. Here, authors construct metal-organic frameworks from polyoxometalates and porphryins to direct electron flow and improve CO2 reduction efficiencies.

## Introduction

The anthropic excessive emission of carbon dioxide (CO_2_) has resulted in serious climate change and environmental crisis, such as global warming, acid rain, and rising of sea level, etc^1^. Powerful methods like storage and conversion of CO_2_ have been developed to reduce atmospheric CO_2_ level^2–4^. In contrast, conversion of CO_2_ is regarded as more promising way, due to which can make CO_2_ transform into serviceable high-valued energy products or chemicals (e.g., CO, CH_4_, HCOOH and C_2_H_5_OH, etc.), that would simultaneously mitigate the greenhouse effect and energy crisis^5–7^. At present, the most representative strategy for implementing this conversion process is electrocatalytic CO_2_ reduction reaction (CO_2_RR), because of its characteristic advantages of easy processing, simple devices, and low energy consumption^8–13^. Nevertheless, the electrochemical conversion of CO_2_ still needs to conquer considerable kinetic barrier required by the intrinsically thermodynamic stability and low electron affinity of the CO_2_ molecule, and the accompanying competitive H_2_ evolution is always hindering the generation of aiming product, resulting in hard to discover suitable electrocatalysts to afford high CO_2_RR efficiency and selectivity^14^.

During past decades, researchers have explored different kinds of materials for CO_2_RR electrocatalysis, such as metals^8,15^, transition metal oxides^9,16^, transition metal chalcogenides^17,18^, metal-free 2D materials^14^, and metal-organic frameworks (MOFs)^19–23^, etc^24–27^, among which MOFs are regarded as ascendant CO_2_RR electrocatalysts owing to their structural merits commonly involve in open Lewis acid metal sites in favor of adsorbing CO_2_ molecule, as well as well-defined catalytic environments suitable for mechanism explanation^28–31^. However, it is a pity that their poor electrical conductivity and electron-donating capability are always the major constraints for MOFs being as efficient electrocatalysts, considering the accomplishment of necessary multiple electron transfer process for obtaining any reductive products in CO_2_RR. Consequently, integrating electron-rich unit, electron mobility and active component related to specifically electrocatalytic CO_2_ reduction product, into MOF will likely to be an effective approach to improve the selectivity and efficiency of CO_2_RR.

As a proof-of-concept, we decided that assembling reductive polyoxometalates (POMs) and metalloporphyrins to construct MOFs so as to verify the feasibility of this inference^32,33^. The corresponding considerations are listed as follows. Firstly, reductive POMs mainly composed of low valent metal ions, such as Zn-ε-Keggin cluster ({ε-PMo_8_^V^Mo_4_^VI^O_40_Zn_4_}^34,35^, including eight Mo^V^ atoms), are usually electron-rich aggregates^36^ and can easily offer electrons when triggered by redox reaction or bias stimulus; secondly, metalloporphyrin, where inherent macrocycle conjugated π-electron system is very beneficial for electron mobility, has proven to be advantageous to produce CO in electrocatalytic CO_2_RR^19,37–39^; thirdly, the connection of POM and metalloporphyrin will presumably create an oriented electron transportation pathway under the motivation of electric field, abundant electrons flowing from POM cluster to metalloporphyrin motif can guarantee and facilitate the fulfillment of multiple electron migration process of CO_2_RR; and finally, polyoxometalate-based MOFs always performing good structural robustness and chemical stability that is essential to the durability test of CO_2_RR. From the above we deduce that reductive polyoxometalate-metalloporphyrin organic frameworks (PMOFs) will probably be promising candidates to enhance the efficiency and selectivity of CO_2_RR, while the assembly of this type of structures is still extremely challenging^40^.

Herein, we synthesize a series of stable PMOFs constructed by tetrakis[4-carboxyphenyl]-porphyrin-M (M-TCPP) linkers and reductive Zn-ε-Keggin clusters through an in situ hydrothermal method with similar formulae of [PMo^V^_8_Mo^VI^_4_O_35_(OH)_5_Zn_4_]_2_[M-TCPP][2H_2_O][1.5TBAOH] (M = Fe, Co, Ni, and Zn). The synergistic combination of Zn-ε-Keggin and M-TCPP in these PMOFs can serve as the role of gathering electron donating, electron migration, and electrocatalytic active component in the CO_2_RR. The electroreduction performances of diverse transition metal (i.e., Co, Fe, Ni, and Zn) based PMOFs are tested and relative results are discussed in the single-crystal level. As expected, these PMOFs show excellent performances in electrocatalytic CO_2_RR, especially for Co-PMOF, which is able to selectively convert CO_2_ to CO with a superior faradaic efficiency of 99% (highest in reported MOFs), a high TOF of 1656 h^−1^ (−0.8 V) and excellent stability for more than 36 h. It is worth noting that this is the first case to explore polyoxometalate-based MOF in the application of electrochemical CO_2_RR.

## Results

### Structure and characterization of M-PMOFs

**Fig. 1:**
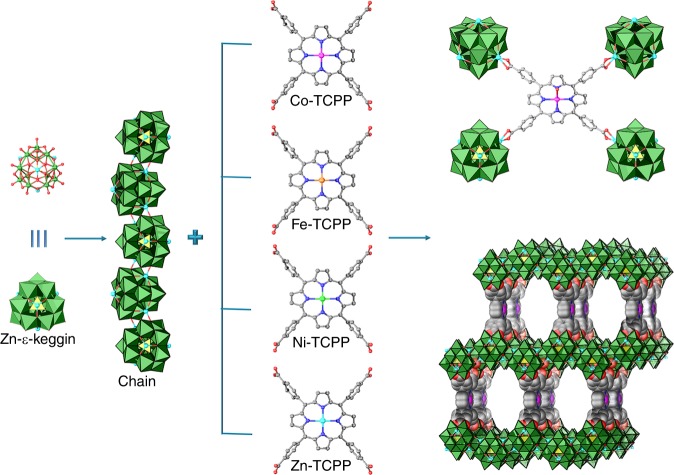
Schematic illustration of the structures of M-PMOFs (M = Co, Fe, Ni, Zn). M-PMOF was constructed by the 4-connected TCPP linkers and zigzag POM chains

M-PMOF (i.e., Co-PMOF, Fe-PMOF, Ni-PMOF, and Zn-PMOF) samples were synthesized under similar hydrothermal conditions. A mixture of M-TCPP, Na_2_MoO_4_·2H_2_O, H_3_PO_3_ and zinc chloride was treated at 180 °C for 72 h and thus-obtained crystals were washed and measured. Single-crystal X-ray diffraction studies reveal that M-PMOF crystals have iso-reticular structures and are crystallized in orthorhombic ***Fmmm*** space group (Fig. 1). In the structure, Zn-ε-Keggin and M-TCPP present tetrahedral and quadrilateral connection modes, respectively. One Zn-ε-Keggin cluster with four Zn (II) centers connects with adjacent Zn-ε-Keggin clusters with two Zn-O bonds to give cluster chain. Chains are further connected by M-TCPP ligands to provide a three-dimensional framework with two-fold interpenetrated ***mog*** topology (Supplementary Fig. 1). Typically, in the structure, four Zn-ε-Keggin chains (adjacent distance is about 15 Å) are linked by M-TCPP ligands through the coordination of Zn (II) and carboxylic groups. The distance between two nearest and parallel M-TCPP units in uninterpenetrated network is about 17 Å (Fig. 1). The interpenetrated topology might endow these structures with high chemical, thermal, or catalysis stability and the direct connection of Zn-ε-Keggin with M-TCPP ligands in a structure would presumably create an oriented electron transportation pathway that might be beneficial for efficient charge transfer.

To investigate their stability, M-PMOF samples are successfully prepared and certified by the powder X-ray diffraction (PXRD) tests (Supplementary Figs. 3–6). The thermal stability of M-PMOF samples is studied by thermogravimetric analysis (TGA) in O_2_ atmosphere with a heating rate of 10 °C min^−1^. Under O_2_ atmosphere, all these M-PMOF samples can be stable at temperatures higher than 200 ^o^C. Taking Co-PMOF for example, about 4% mass loss at temperature range from 0 to 100 ^o^C is attributed to the loss of guest molecules. After 200 °C, the framework of Co-PMOF starts to collapse and ends at about 500 °C (Supplementary Fig. 8). To further certify the thermal stability, PXRD patterns of M-PMOF samples treated at 200 °C in the presence of ultrapure O_2_ were tested. As shown in Supplementary Fig. 9, the PXRD patterns of M-PMOF samples still agree well with the simulated ones. Furthermore, M-PMOF samples show high chemical stability. All the M-PMOF samples enable to be stable in KHCO_3_ aqueous (0.5 M) for more than 24 h, guaranteeing the further investigation of electroreduction performance (Supplementary Figs. 10–13). Moreover, after 24 h test, all of them can maintain their structures at pH range from 5 to 11 proved by PXRD tests, IR, and mapping test, respectively (Supplementary Figs. 14, 15). The high chemical and thermal stability of M-PMOF samples set fundamental basis for further applications.

### The electrocatalytic performance of M-PMOFs

The electrochemical CO_2_RR performance is evaluated in an airtight three electrodes electrochemical H-type cell (the compartments are separated by a proton exchange membrane (Nafion®212)). All the M-PMOFs (i.e., Co-PMOF, Fe-PMOF, Ni-PMOF, and Zn-PMOF) samples are packed in the cells and tested. In this work, all the potentials are measured vs. Ag/AgCl electrode and the results are reported vs. reversible hydrogen electrode (RHE).

**Fig. 2:**
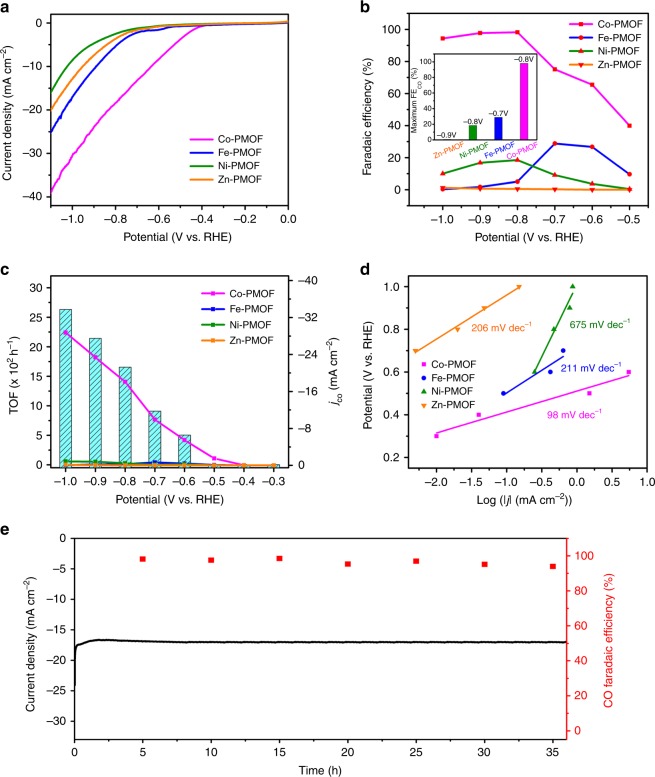
Electrocatalytic performances of M-PMOFs. **a** Linear sweep voltammetric curves. **b** Faradaic efficiencies for CO. **c** Partial CO current density and TOFs. **d** Tafel plots. **e** Durability test of Co-PMOF at the potential of −0.8 V vs. RHE. These tests are performed in 0.5 M KHCO_3_ electrolyte and at the scan rate of 5 mV s^−1^

Linear sweep voltammetry (LSV) curves (without *iR* compensation) show that Co-PMOF has a small onset potential of −0.35 V and is much more positive than that of Fe-PMOF (−0.53 V), Ni-PMOF (−0.58 V), and Zn-PMOF (−0.60 V) in CO_2_-saturated 0.5 M KHCO_3_ (pH = 7.2) solution (Fig. 2a). Co-PMOF exhibits a small overpotential of 0.24 V and the relative equilibrium potential of CO_2_/CO is −0.11 V. Besides, Co-PMOF presents a total current density of 38.9 mA cm^−2^ at −1.1 V and is superior to Fe-PMOF (25.1 mA cm^−2^), Ni-PMOF(20.02 mA cm^−2^), and Zn-PMOF (16 mA cm^−2^). Due to its excellent performances, this work will mainly focus on Co-PMOF to discuss the possible relations between its properties and well-defined structure.

Combing POMs and M-TCPP as building units, the high electrochemical activity of Co-PMOF is supposed to come from CO_2_RR. To prove it, relative tests are conducted both in N_2_ and CO_2_ saturated KHCO_3_ solution. As expected, Co-PMOF exhibits a more negative onset potential and a smaller current density in N_2_-saturated KHCO_3_ solution than that in CO_2_-staurated KHCO_3_ solution (Supplementary Fig. 16). Furthermore, the GC analyses show that H_2_ and CO are the primary reduction products and there is no liquid product detected by ^1^H nuclear magnetic resonance spectroscopy (Supplementary Fig. 17). To further evaluate the selectivity of M-PMOF samples for CO_2_RR, the corresponding FE for CO and H_2_ (denoted as FE_CO_ and FE_H2_) are calculated over the entire potential range (Fig. 2b and Supplementary Fig. 18). Taking Co-PMOF for example, the initial formation of CO is detected by GC at the potential of −0.35 V with a small CO partial current density of 0.39 mA cm^−2^. With the increase of potential, the FE_CO_ continuously increases and reaches up to a maximum value of 98.7% at −0.8 V and keeps higher than 94% even at −1.0 V (Supplementary Fig. 19). The high FE_CO_ over such as wide potential range (from −0.8 V to −1.0 V) outperforms many reported materials (Supplementary Table 1)^41–43^. Noteworthy, the maximum FE_CO_ value (98.7%) of Co-PMOF is higher than other M-PMOFs (i.e., Fe-PMOF, 28.8%; Ni-PMOF, 18.5%, and Zn-PMOF, 1.2%, respectively) and is highest among reported MOFs (Supplementary Table 1)^36,37^. In addition, the same electrochemical experiment of Co-PMOF is carried out in N_2_-saturated 0.5 M KHCO_3_ to reveal the source of CO and there is no CO detected under this condition (Supplementary Fig. 21). Besides, an isotopic experiment that using ^13^CO_2_ as substrate was performed under identical reaction conditions to reveal the carbon source of CO. The products were analyzed by gas chromatography and mass spectra. As shown in Supplementary Fig. 22, the peak at *m/z* = 29 is assigned to ^13^CO, indicating that the carbon source of CO indeed derives from the CO_2_ used.

To further reveal the efficiency of the remarkable performances of Co-PMOF, partial current densities of CO and H_2_ at different potentials are detected (Fig. 2c and Supplementary Fig. 23). Co-PMOF gives a partial CO current density of 18.08 mA cm^−2^ at −0.8 V. This value is 30 times larger than that of Fe-PMOF (0.27 mA cm^−2^), Ni-PMOF (0.47 mA cm^−2^), and Zn-PMOF (0.02 mA cm^−2^). Besides, TOF of Co-PMOF is calculated to be 1656 h^−1^ at −0.8 V and the TON values of Co-PMOF following the change of time was shown in Supplementary Fig. 24^37^. To the best of our knowledge, this catalytic behavior of Co-PMOF outperforms most of MOF catalysts and is one of the best reported materials (Supplementary Table 1)^43,44^.

To elucidate the dynamics activity of Co-PMOF for electrochemical CO_2_RR, Tafel slopes are calculated based on Tafel equation (*η* = b log *j* + a, where *η* is the overpotential, *j* is the current density and b is the Tafel slope) and presented in Fig. 2d. Remarkably, the Tafel slope for Co-PMOF is 98 mV dec^−1^, which is much smaller than that of Fe-PMOF (211 mV dec^−1^), Ni-PMOF (675 mV dec^−1^), and Zn-PMOF (206 mV dec^−1^). This indicates the favorable kinetics of Co-PMOF for the formation of CO, which might be ascribed to the more efficient charge transfer and larger active surface in the catalytic process. To support the hypothesis, electrochemical double-layer capacitance (*C*_*dl*_) is calculated to estimate the electrochemical active surface area (ECSA) to further discuss the potential influence factors (Supplementary Fig. 25)^45^. The result show that Co-PMOF indeed exhibits larger *C*_*dl*_ value (12.17 mF cm^−2^) than that of Fe-PMOF (10.26 mF cm^−2^), Ni-PMOF (10.16 mF cm^−2^), and Zn-PMOF (9.83 mF cm^−2^), which enable to provide more active sites in electrocatalyst to contact the electrolyte for being beneficial to increase the reaction speed of electrochemical CO_2_RR.

Moreover, electrochemical impedance spectroscopy (EIS) measurement is carried out to probe the electrocatalytic kinetics on the electrode/electrolyte surface. The Nyquist plots demonstrate that Co-PMOF has much smaller charge transfer resistance (9.83 Ω) than Fe-PMOF (10.26 Ω), Ni-PMOF (10.70 Ω), and Zn-PMOF (12.17 Ω) during the electrochemical CO_2_RR (Supplementary Fig. 26). This indicates Co-PMOF can provide faster electron transfer from the catalyst surface to the reactant (i.e., CO_2_) in intermediate (HCOO* and CO*) generation, eventually resulting in largely enhanced activity and selectivity. This conclusion will be further verified by the theoretical calculations and relative contents are discussed in the mechanism part.

Stability is a key factor to evaluate the durability properties of catalysts. To evaluate the CO_2_RR stability of Co-PMOF, long-time durability test is performed with chronoamperometric test at a fixed overpotential of 0.69 V in 0.5 M KHCO_3_ solution (Fig. 2e). After 36 h, negligible decay in activity and FE_CO_ is detected (the gaseous product is analyzed by GC every 5 h). During the process, the corresponding FE_CO_ can be retained at values more than 90% and the current density is about 17 mA cm^−2^ over the entire experiment. This stability measurement reveals that Co-PMOF is highly stable electrocatalyst, which has much potential to be used in efficient electrochemical CO_2_RR (Supplementary Figs. 27, 38).

## Discussion

**Fig. 3:**
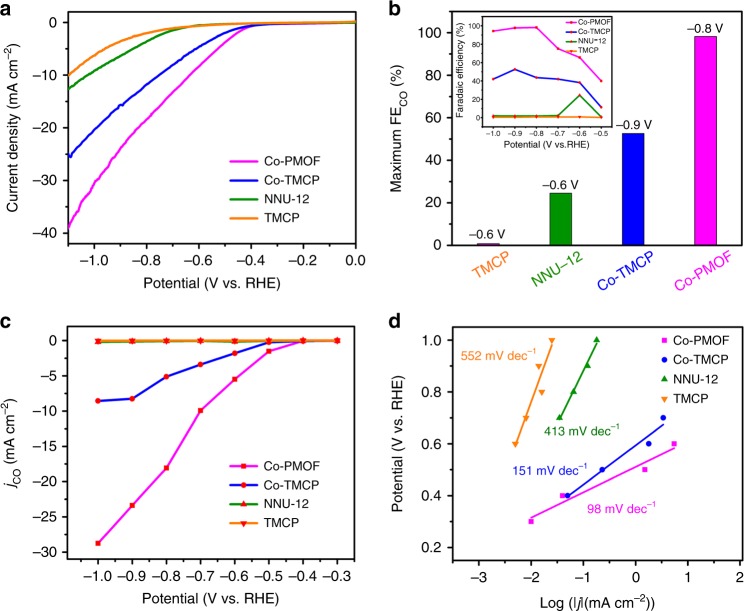
Electrocatalytic performances of contrast samples for Co-PMOF. **a** Linear sweep voltammetric curves. **b** Maximum FE_CO_ of TMCP (−0.6 V), NNU−12 (−0.6 V), Co-TMCP (−0.9 V) and Co-PMOF (−0.8 V). **c** Partial CO current density. **d** Tafel plots

To reveal the catalytic mechanism, a series of contrast samples are prepared and tested. The electrochemical CO_2_RR of contrast samples are measured adopting the same testing methods. To evaluate the impact of Co-TCPP in electrochemical CO_2_RR, NNU-12 {[PMo_8_^V^Mo_4_^VI^O_36_(OH)_4_Zn_4_][BCPT]_2_∙xGuests} is prepared^46^. NNU-12 with ***dia*** topology is constructed by Zn-ε-Keggin and BCPT^2-^ linker, which contains the same Zn-ε-Keggin unit as Co-PMOF while the ligand is different. NNU-12 shows poor current density (3.63 mA cm^−2^ at −0.8 V), low FE_CO_ (1.80% at −0.8 V), high onset potential (−0.6 V) with a Tafel slope of 413 mV dec^−1^, and has negligible CO_2_ reduction activity (Fig. 3, Supplementary Figs. 28, 30). This indicates the high CO_2_ reduction activity of Co-PMOF originates from Co-TCPP. In the process, Co-TCPP might act as electrocatalytic center and redox-hopping-based conduit, which is beneficial for charge transfer.

The influence of reductive POM in the electrochemical CO_2_RR is also investigated. Tetramethyl 5, 10, 15, 20-tetrakis[4-carboxyphenyl]-porphyrin-Co (Co-TMCP) is prepared and shows a small onset potential of −0.4 V, high current density (11.60 mA cm^−2^ at −0.8 V) with a Tafel slope of 151 mV dec^−1^ (Supplementary Fig. 29 and Fig. 3). In contrast, Co-TMCP exhibits poorer CO_2_ reduction performance than Co-PMOF, which might be attributed to the lower proton and electron transfer efficiency (Fig. 3 and Supplementary Fig. 30). Zn-ε-Keggin with strong reducibility might provide additional electrons and facilitate the charge transfer for Co-PMOF in electrochemical CO_2_RR.

In addition, tetramethyl 5, 10, 15, 20-tetrakis[4-carboxyphenyl]-porphyrin (TMCP) is prepared to study the effect of Co in the reduction process. TMCP shows poorer current density (2.06 mA cm^−2^ at −0.8 V), lower FE_CO_ (0.77% at −0.8 V), higher onset potential (−0.67 V) with a Tafel slope of 552 mV dec^−1^ and has no CO_2_RR activity than Co-PMOF (Fig. 3, Supplementary Figs. 29, 30), implying Co is the electrochemically active site in Co-PMOF. To further support the results, ECSA and EIS measurements are conducted and relative results are calculated. These results give similar performance tendency in Co-PMOF as discussed above (Supplementary Fig. 34 and 35). Moreover, MOF-525(Co) was prepared as relevant comparison to further support the catalytic mechanism of M-PMOF (Supplementary Fig. 31). MOF-525(Co) has similar ligand as Co-PMOF and is constructed with Zr_6_O_4_(OH)_4_ unit without POM^47^. MOF-525(Co) is successfully prepared and certified by the PXRD tests (Supplementary Fig. 32). The electrochemical CO_2_RR of MOF-525(Co) is measured adopting the same testing methods. As shown in Supplementary Fig. 33, MOF-525(Co) shows lower FE_CO_ (47.9% at −0.8 V) than Co-PMOF (98.7% at −0.8 V), which might be attributed to the poorer proton and electron transfer efficiency. This indicates POM actually acts as electron-rich aggregates in the catalytic mechanism of M-PMOF.

**Fig. 4:**
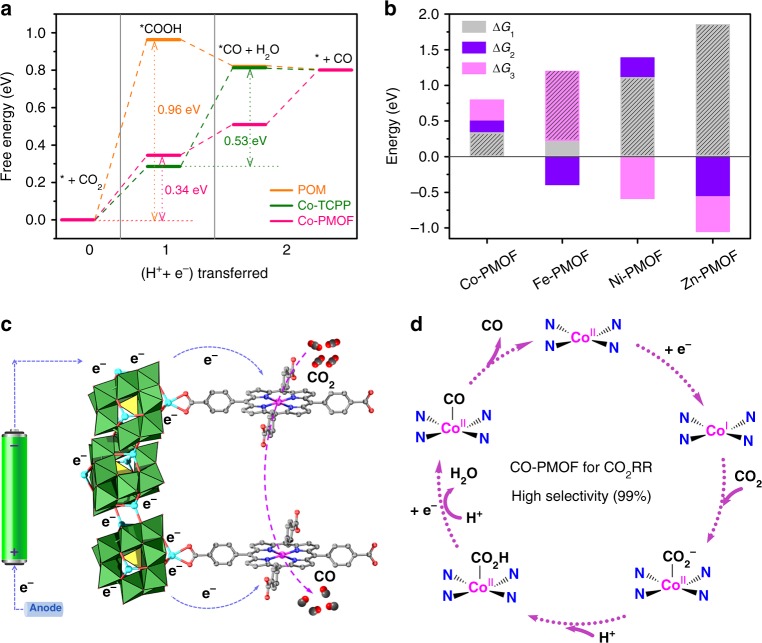
The DFT calculation and proposed reaction mechanism. **a** The free energy diagrams of CO_2_ reduction to CO for POM (orange), Co-TCPP (green), and Co-PMOF (pink). The rate-determining step and the corresponding free energy on each material are indicated (Note that asterisk represents a surface active site for reaction). **b** Comparison of the free energy of each elementary reaction (Δ*G*_1_, Δ*G*_2_, and Δ*G*_3_ represent the free energy of *COOH formation, *CO formation, and CO desorption process, respectively) in CO_2_RR for Co-PMOF, Fe-PMOF, Ni-PMOF, and Zn-PMOF, respectively. **c**, **d** Proposed mechanistic scheme for the CO_2_RR on Co-PMOF (POM as electron-donating group, porphyrins as charge transfer ligand and transition metals as electron-collecting group)

To understand the highly active as well as selective reaction mechanism of Co-PMOF, DFT calculations are performed (Supplementary note 2). Generally, the electroreduction of CO_2_ to CO includes three elementary reactions, the formation of *COOH and *CO with one electron transfer for each of them, and the finally CO desorption process. The asterisk (*) represents the surface active sites for adsorption and reaction. From the calculated free energy diagrams shown in Fig. 4a, the rate-determining steps (RDS) for CO_2_ reduction on single Zn-ε-Keggin (POM) and Co-TCPP are the formation of adsorbed intermediates *COOH and *CO with rather high free energies of Δ*G*_1_ = 0.96 eV and Δ*G*_2_ = 0.53 eV, respectively. Expectedly, when assembling the Zn-ε-Keggin and Co-TCPP together, the final compound of Co-PMOF possesses remarkably reduced Δ*G*_1_ and Δ*G*_2_, and particularly the RDS of *COOH formation decrease to a much smaller free energy of Δ*G*_1_ = 0.34 eV, being consistent with the high electroreduction activity and selectivity of this PMOF. These results indicate not only the favorable active site is the Co in Co-TCPP instead of POM, but also the efficiently synergistic electron modulation of POM and the porphyrin metal center. Then, the reaction pathways are further calculated by replacing the Co in porphyrin center with Fe, Ni, and Zn (Fig. 4b and Supplementary Fig. 36). For Fe-PMOF, although the energy of *COOH and *CO formations have decreased when compared to that of Co-PMOF, the desorption of CO become harder (Δ*G*_3_ = 0.98 eV), which may be ascribed to the higher affinity of Fe. For the case of Ni and Zn, the RDS both are the formation of *COOH with much higher free energies of 1.12 and 1.86 eV, respectively. This indicates the initial proton-coupled electron transfer to CO_2_ forming an adsorbed *COOH intermediate is sluggish in these systems. In a word, the sequence of the reaction energies on different metal centers is well consistent with our experiments, which further confirms the high activity of the Co modified compounds and the modulation effect in the M-PMOF systems.

Based on the experiment results and theoretical calculations, we elaborate the reduction processes from CO_2_ to CO on Co-PMOF and the possible mechanism is summarized in Fig. 4d and Supplementary note 3. During the electrochemical process, Co centers are reduced from Co(II) to Co(I) proved by the cyclic voltammetry (CV) tests (Supplementary Fig. 37), which has been detected in many Co-centered macrocycles^11,29,37,48^. As proposed in the mechanism, Zn-ε-Keggin initially traps the electron from the electrodes and transfers it to the Co center (Fig. 4c). Meanwhile, the Co center is reduced from Co(II) to Co(I) during the process. Then Co(I) interacts with carbon monoxide to give Co(II)*COOH intermediate coupling with the proton-electron transfer. Subsequently, Co(II)*COOH convert to Co(II)*CO. Finally, CO is desorbed and produced (Fig. 4d).

In summary, we have designed and synthesized a series of stable PMOFs by applying reductive Zn-ε-Keggin cluster and metalloporphyrin as building block and linker, respectively. The direct communication of reductive POM unit and metalloporphyrin under the exertion of the electric field results in an oriented electronic transportation channel that is extremely conductive to the accomplishment of multiple electrons transfer process in electrocatalytic CO_2_RR. Obviously, these PMOFs show excellent electrochemical CO_2_ reduction performances at last. In particular for Co-PMOF, the best one of all target compounds, enables to selectively convert CO_2_ to CO with a remarkable faradaic efficiency of 99% (highest in reported MOFs), a high TOF of 1656 h^−1^ (−0.8 V) and excellent stability for more than 36 h. Specially, DFT calculation demonstrates the superior catalysis performances of Co-PMOF than other transition metals (i.e., Fe, Ni, and Zn) and further reveals the synergistic effect of reductive POM and Co-porphyrin. This strategy opens great perspectives in designing novel and efficient CO_2_RR electrocatalysts and might shed light on the exploration of powerful protocols to address CO_2_ problems.

## Methods

### Syntheses of various M-TMCPs

5, 10, 15, 20-Tetrakis(4-methoxycarbonylphenyl)porphyrin (TMCP) (1.7 g, 2.0 mmol), CoCl_2_·6H_2_O (6.1 g, 25.6 mmol), and DMF (200 mL) were mixed and refluxed for 12 h. A large amount of water was added after cooling down to room temperature. The as-synthesized material was filtered and washed with water (600 ml) for six times. The fuchsia solid sample was obtained (yield 90% based on TMCP). The preparation processes of Fe-TMCP, Ni-TMCP, and Zn-TMCP were similar to Co-TMCP except that CoCl_2_·6H_2_O (6.1 g, 25.6 mmol) was replaced by FeCl_2_·4H_2_O (5.0 g, 25.6 mmol), NiCl_2_·6H_2_O (6.2 g, 25.6 mmol), and ZnCl_2_ (3.5 g, 25.6 mmol), respectively^49^.

### Syntheses of various M-TCPPs

The obtained ester, MeOH (50 mL) and THF (50 mL) were mixed and stirred. A volume of 50 mL KOH solution (5.2 g, 93.9 mmol KOH was dissolved in 50 mL H_2_O) was added into the mixture and was refluxed for one night. After reaction, the organic solvents were evaporated. To make the solid fully dissolve, moderate water was added to the mixed solution and heated. 2 M HCl was used to acidify the obtained fuchsia homogeneous solution until no further fuchsia precipitate was generated. The solid was collected and washed with deionized water until the pH of filtrate reaches about 5. Finally, the solid sample was dried in vacuum at 60 °C. The colors of Co-TCPP, Fe-TCPP, and Ni-TCPP were red, brown, and crimson, respectively^49^.

### Syntheses of various M-PMOFs

Taking Co-PMOF for example, a mixture of H_3_PO_3_ (10 mg, 0.125 mmol), zinc chloride (68 mg, 0.50 mmol), Na_2_MoO_4_·2H_2_O (310 mg, 1.28 mmol), and tetrabutylammonium hydroxide (TBAOH) solution (250 μL, 10 wt % in water) was added into 3.5 mL H_2_O and stirred for 15 min. 2 mol L^−1^ HCl solution was used to adjust the pH of the mixture solution until the pH reached about 5. Then, 5,10,15,20-tetrakis(4-carboxyphenyl)porphyrinato-Co (Co-TCPP) (132 mg), Mo powder 99.99% (25 mg), and dimethylacetamide solvent (250 μL) were added into the mixed solution and stirred in a 15 mL Teflon-lined stainless steel autoclave and heated at 180 °C for 72 h. After temperature cooled down to room temperature with a rate of 15 °C h^−1^, the crystals were washed with water and collected (yield was about 75% based on Co-TCPP). The preparation processes of Ni-PMOF and Fe-PMOF were similar to Co-PMOF except that Co-TCPP (132 mg) was replaced by Ni-TCPP (132 mg) and Fe-TCPP (33 mg), respectively. It is worthy to note that the preparation process of Zn-PMOF was similar to Co-PMOF except that Co-TCPP (132 mg) is replaced by TCPP (118 mg) (Supplementary Fig. 2). The IR peaks are listed as follows (Supplementary Fig. 7, KBr pellets, ν cm^−1^): Co-PMOF: 3427 (s), 3122 (w), 2958 (w), 1699 (w), 1604 (s), 1400 (s), 1040 (m), 1271 (w), 1172 (w), 1027 (m), 941 (s), 869 (w), 796 (s), 713 (m), 594 (w), 495 (w); Fe-PMOF: 3446 (s), 3211 (w), 1701 (w), 1602 (s), 1404 (s), 1271 (w), 1043 (m), 931 (s), 864 (w), 783 (m), 714 (m), 595 (w), 489 (w); Ni-PMOF: 3436 (s), 3214 (w), 1704 (w), 1606 (s), 1399 (s), 1261 (w), 1041 (m), 930 (s), 861 (w), 785 (m), 712 (m), 597 (w), 490 (w) and Zn-PMOF: 3431 (s), 3188 (w), 1703 (w), 1600 (s), 1401 (s), 1272 (w), 1038 (m), 937 (s), 865 (w), 789 (m), 711 (m), 596 (w), 492 (w). For the formulae of M-PMOF, all of these M-PMOF are iso-reticular in structure and only have minor difference in centered metal ions. Their structures have been well-defined in the SXRD tests. Based on the elemental analyses and SXRD tests, the calculated formulae was [PMo^V^_8_Mo^VI^_4_O_35_(OH)_5_Zn_4_]_2_[M-TCPP][2H_2_O][1.5TBAOH] (M = Fe, Co, Ni, and Zn). This result was further supported by TGA test (Supplementary Fig. 8).

### Synthesis of MOF-525

ZrCl_4_·8H_2_O (12.5 mg, 0.037 mmol) and DMF (10 mL) were sonicated for 30 min. Then, tetrakis(4-carboxyphenyl)porphyrin (2.5 mg, 0.037 mmol) was added into the solution. After sonication for 10 min, acetic acid (2.5 mL) was added. The solution was placed in a 20 mL scintillation vial and heated at 65 °C for 3 days. The microcrystalline powder was filtered and washed with DMF, acetone for several times. Finally, the powder was dried in vacuum at 120 °C for 48 h^47^.

### Synthesis of MOF-525(Co)

CoCl_2_·6H_2_O (100 mg, 0.62 mmol), MOF-525 (50 mg), and DMF (10 mL) were mixed and heated at 100 °C for 18 h. The microcrystalline powder were collected by filtration and washed with DMF, acetone for several times. Finally, the powder was dried in vacuum at 120 °C for 48 h^47^.

### Characterizations and instruments

Powder X-Ray diffraction (PXRD) patterns of samples were carried out on a D/max 2500 VL/PC diffractometer (Japan) equipped with Cu Kα radiation (λ = 1.54060 Å). FTIR spectra were tested on a Bruker Tensor 27 FT/IR spectrophotometer. Thermogravimetric analyses of crystal samples were measured on a Netzsch STA449F3 analyzer under the oxygen atmosphere with a heating rate of 10 °C min^−1^. Single-crystal XRD data of Co-PMOF, Fe-PMOF, Ni-PMOF, and Zn-PMOF were measured on a Bruker APEXII CCD diffractometer with graphite-monochromated Mo Kα radiation (λ = 0.71073 Å) at 296 K. All the structures were solved by direct method using SHELXT program and refined on an Olex^2^ software. Multi-scan technique was used to absorption corrections. The TBA^+^ ion could not be detected in the structure and some other guest molecules were defined as disorder. The detailed crystallographic information of these crystals was listed in Supplementary Table 2. The CCDC number of Co-PMOF, Fe-PMOF, Ni-PMOF, and Zn-PMOF (NNU-13) were 1832596 [10.5072/ccdc.csd.cc1zjbmg], 1832597 [10.5072/ccdc.csd.cc1zjbnh], 1832598 [10.5072/ccdc.csd.cc1zjbpj], and 1181860 [10.5072/ccdc.csd.cc1ytd38] respectively.

### Electrochemical measurements

All electrocatalysis tests of the catalysts were performed at ambient environment on the electrochemical workstation (Bio-Logic) in a standard three-electrode configuration in 0.5 M KHCO_3_ (pH = 7.2). Pt wire and Ag/AgCl electrode were used as the counter and reference electrode, respectively. The working electrode was modified carbon cloth (1 cm × 1 cm). The experiment was performed in an airtight electrochemical H-type cell with a catalyst-modified carbon cloth electrode (denoted as CCE, 1 cm × 2 cm) as the work electrode.

Given the poor intrinsic electrical conductivity of MOFs, acetylene black (AB) was introduced to mix with the as-synthesized MOFs to improve the conductivity. Nafion solution was introduced as a kind of MOF dispersion solution generally applied in many reported works, which can form a homogeneous ink with MOF and further help to attach onto the surface of carbon cloth. The pure carbon cloth has no CO_2_RR activity (Supplementary Fig. 20). The preparation of the CCE working electrode was as follows. A volume of 10 mg electrocatalyst, 10 mg acetylene black (AB), and 1 mL 0.5% Nafion solution were grounded to form uniform catalyst ink. After sonication for 30 min, the ink was dropped directly onto a carbon cloth (1 cm × 1 cm) with a catalyst loading density of ~1 mg cm^−2^ and dried. In the H-type cell, two compartments were separated by an exchange membrane (Nafion®212).

During the CO_2_ reduction experiments, the polarization curves were performed by LSV mode at a scan rate of 5 mV s^−1^. Initially, polarization curves for the modified electrode were recorded under an inert N_2_ (gas) atmosphere. After that, the solution was bubbled with CO_2_ (99.999%) for at least 30 min to make the aqueous solution saturated and then the electrocatalytic CO_2_RR was conducted (Supplementary note 1). Potential was measured vs. Ag/AgCl electrode and the results were reported vs. reversible hydrogen electrode based on the Nernst equation: E (vs. RHE) = E (vs. Ag/AgCl) + 0.1989 V + 0.059 × pH.

EIS measurement was carried out by applying an AC voltage with 10 mV amplitude in a frequency range from 1000 kHz to 100 mHz at a overpotential of −0.8 V (vs. RHE). To estimate the ECSA, cyclic voltammograms (CV) were tested by measuring double-layer capacitance (*C*_*dl*_) under the potential window of −0.45 V to −0.55 V (vs. Ag/AgCl) with various scan rates from 10 to 100 mV s^−1^. All the LSV curves were presented without iR compensation.

## Electronic supplementary material

Supplementary Information

Peer Review File

## Data Availability

The data that support the findings of this study are available from the corresponding author upon reasonable request.

## References

[1] Shakun JD (2012). Global warming preceded by increasing carbon dioxide concentrations during the last deglaciation. Nature.

[2] Rao H, Schmidt LC, Bonin J, Robert M (2017). Visible-light-driven methane formation from CO_2_ with a molecular iron catalyst. Nature.

[3] De Luna P (2018). Catalyst electro-redeposition controls morphology and oxidation state for selective carbon dioxide reduction. Nat. Catal..

[4] Haas T (2018). Technical photosynthesis involving CO_2_ electrolysis and fermentation. Nat. Catal..

[5] Wu JH, Huang Y, Ye W, Li YG (2017). CO_2_ reduction: from the electrochemical to photochemical approach. Adv. Sci..

[6] Diercks CS (2018). Reticular electronic tuning of porphyrin active sites in covalent organic frameworks for electrocatalytic carbon dioxide reduction. J. Am. Chem. Soc..

[7] Zhang X (2017). Highly selective and active CO_2_ reduction electrocatalysts based on cobalt phthalocyanine/carbon nanotube hybrid structures. Nat. Commun..

[8] Li Q (2017). Tuning Sn-catalysis for electrochemical reduction of CO_2_ to CO via the core/shell Cu/SnO_2_ structure. J. Am. Chem. Soc..

[9] Gao S (2016). Partially oxidized atomic cobalt layers for carbon dioxide electroreduction to liquid fuel. Nature.

[10] Jiao Y (2017). Molecular scaffolding strategy with synergistic active centers to facilitate electrocatalytic CO_2_ reduction to hydrocarbon/alcohol. J. Am. Chem. Soc..

[11] Lin S (2015). Covalent organic frameworks comprising cobalt porphyrins for catalytic CO_2_ reduction in water. Science.

[12] Seh ZW (2017). Combining theory and experiment in electrocatalysis: insights into materials design. Science.

[13] Duan XC (2017). Metal-free carbon materials for CO_2_ electrochemical reduction. Adv. Mater..

[14] Sun ZY (2017). Fundamentals and challenges of electrochemical CO_2_ reduction using two-dimensional materials. Chem.

[15] Zhang WJ (2018). Progress and perspective of electrocatalytic CO_2_ reduction for renewable carbonaceous fuels and chemicals. Adv. Sci..

[16] Zhang L, Zhao ZJ, Gong JL (2017). Nanostructured materials for heterogeneous electrocatalytic CO_2_ reduction and their related reaction mechanisms. Angew. Chem. Int. Ed..

[17] Asadi M (2016). Nanostructured transition metal dichalcogenide electrocatalysts for CO_2_ reduction in ionic liquid. Science.

[18] Zhu DD, Liu JL, Qiao SZ (2016). Recent advances in inorganic heterogeneous electrocatalysts for reduction of carbon dioxide. Adv. Mater..

[19] Liao PQ, Shen JQ, Zhang JP (2017). Metal-organic frameworks for electrocatalysis. Coord. Chem. Rev..

[20] Weng Z (2016). Electrochemical CO_2_ reduction to hydrocarbons on a heterogeneous molecular Cu catalyst in aqueous solution. J. Am. Chem. Soc..

[21] Maurin A, Robert M (2016). Noncovalent immobilization of a molecular iron-based electrocatalyst on carbon electrodes for selective, efficient CO_2_-to-CO conversion in water. J. Am. Chem. Soc..

[22] Hinogami R (2012). Electrochemical reduction of carbon dioxide using a copper rubeanate metal organic framework. ECS Electrochem. Lett..

[23] Kumar RS, Kumar SS, Kulandainathan MA (2012). Highly selective electrochemical reduction of carbon dioxide using Cu based metal organic framework as an electrocatalyst. Electrochem. Commun..

[24] Weng Z (2018). Active sites of copper-complex catalytic materials for electrochemical carbon dioxide reduction. Nat. Commun..

[25] Li X (2017). Exclusive Ni-N_4_ sites realize near-unity CO selectivity for electrochemical CO_2_ reduction. J. Am. Chem. Soc..

[26] Wang XQ (2018). Regulation of coordination number over single Co sites: triggering the efficient electroreduction of CO_2_. Angew. Chem. Int. Ed..

[27] Guo SJ (2017). A Co_3_O_4_-CDots-C_3_N_4_ three component electrocatalyst design concept for efficient and tunable CO_2_ reduction to syngas. Nat. Commun..

[28] Liang Z (2017). Pristine metal-organic frameworks and their composites for energy storage and conversion. Adv. Mater..

[29] Kornienko N (2015). Metal-organic frameworks for electrocatalytic reduction of carbon dioxide. J. Am. Chem. Soc..

[30] Hod I (2015). Fe-porphyrin-based metal-organic framework films as high-surface concentration, heterogeneous catalysts for electrochemical reduction of CO_2_. ACS Catal..

[31] Kung CW (2017). Copper nanoparticles installed in metal-organic framework thin films are electrocatalytically competent for CO_2_ reduction. ACS Energy Lett..

[32] Qin JS (2015). Ultrastable polymolybdate-based metal organic frameworks as highly active electrocatalysts for hydrogen generation from water. J. Am. Chem. Soc..

[33] Du DY (2014). Recent advances in porous polyoxometalate-based metal-organic framework materials. Chem. Soc. Rev..

[34] Marleny Rodriguez-Albelo L (2009). Zeolitic polyoxometalate-based metal-organic frameworks (Z-POMOFs): computational evaluation of hypothetical polymorphs and the successful targeted synthesis of the redox-active Z-POMOF1. J. Am. Chem. Soc..

[35] Nohra B (2011). Polyoxometalate-based metal organic frameworks (POMOFs): structural trends, energetics, and high electrocatalytic efficiency for hydrogen evolution reaction. J. Am. Chem. Soc..

[36] Vasilopoulou M (2015). Old metal oxide clusters in new applications: spontaneous reduction of Keggin and Dawson polyoxometalate layers by a metallic electrode for improving efficiency in organic optoelectronics. J. Am. Chem. Soc..

[37] Han N (2017). Supported cobalt polyphthalocyanine for high-performance electrocatalytic CO_2_ reduction. Chem.

[38] Yang XL (2012). Porous metalloporphyrinic frameworks constructed from metal 5,10,15,20-tetrakis(3,5-biscarboxylphenyl)porphyrin for highly efficient and selective catalytic oxidation of alkylbenzenes. Cheminform.

[39] Xie MH, Yang XL, Wu CD (2011). A metalloporphyrin functionalized metal-organic framework for selective oxidization of styrene. Chem. Commun..

[40] Zou C (2012). A multifunctional organic-inorganic hybrid structure based on Mn(III)-porphyrin and polyoxometalate as a highly effective dye scavenger and heterogenous catalyst. J. Am. Chem. Soc..

[41] Albo J (2016). Copper-based metal-organic porous materials for CO_2_ electrocatalytic reduction to alcohols. ChemSusChem.

[42] Kang X (2015). Highly efficient electrochemical reduction of CO_2_ to CH_4_ in ionic liquid using metal-organic framework cathode. Chem. Sci..

[43] Ye L (2016). Highly oriented MOF thin film-based electrocatalytic device for the reduction of CO_2_ to CO exhibiting high faradic efficiency. J. Mater. Chem. A.

[44] Pan Y (2018). Design of single-atom Co-N_5_ catalytic site: a robust electrocatalyst for CO_2_ reduction with nearly 100% CO selectivity and remarkable stability. J. Am. Chem. Soc..

[45] Lei F (2016). Metallic tin quantum sheets confined in graphene toward high-efficiency carbon dioxide electroreduction. Nat. Commun..

[46] Huang Q (2017). A highly stable polyoxometalate-based metal-organic framework with π-π stacking for enhancing lithium ion battery performance. J. Mater. Chem. A.

[47] Morris W (2012). Synthesis, structure, and metalation of two new highly porous zirconium metal–organic frameworks. Inorg. Chem..

[48] Sende JAR (1995). Electrocatalysis of CO_2_ reduction in aqueous media at electrodes modified with electropolymerized films of vinylterpyridine complexes of transition metals. Inorg. Chem..

[49] Feng DW (2012). Zirconium-metalloporphyrin PCN-222: mesoporous metal-organic frameworks with ultrahigh stability as biomimetic catalysts. Angew. Chem. Int. Ed..

