# Supplementary material for: Oriented electron transmission in polyoxometalate-metalloporphyrin organic framework for highly selective electroreduction of CO2
Source: Nat Commun. 2018 Oct 26;9:4466. doi: 10.1038/s41467-018-06938-z (PMC6203756; doi:10.1038/s41467-018-06938-z)
Supplement: Supplementary file 1 — Supplementary Information [file 41467_2018_6938_MOESM1_ESM.pdf]

Supplementary Information for

Oriented Electron Transmission in Polyoxometalate-Metalloporphyrin Organic Framework for Highly Selective Electroreduction of CO_2

Wang et al.

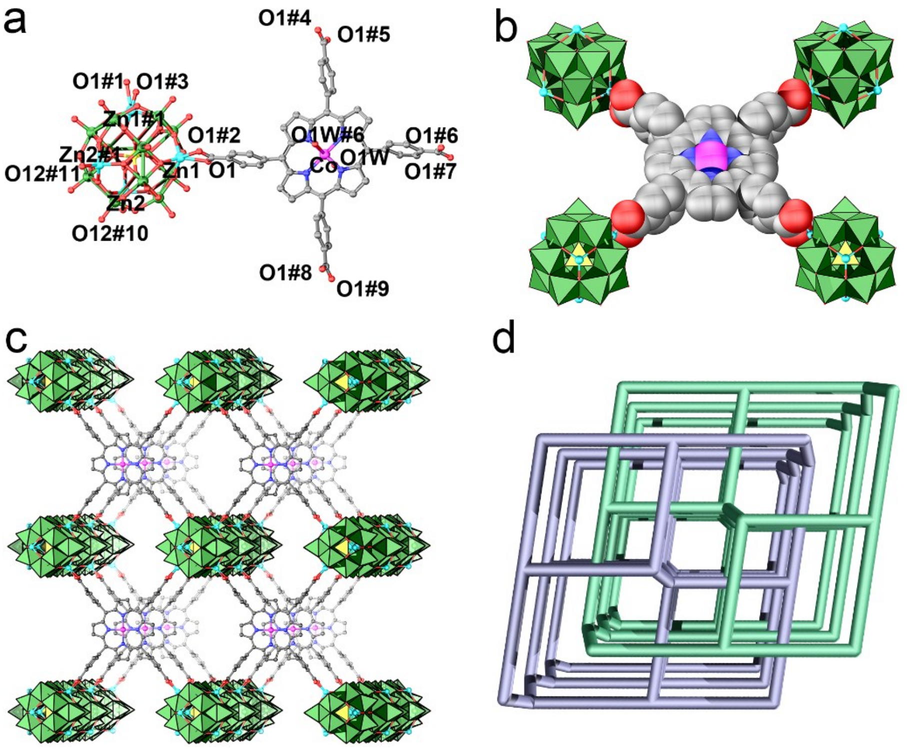

Supplementary Figure 1. Summary of the structure of Co-PMOF. **a** The coordination environments of Zn(II) centers in Co-PMOF. Color code: C, black; N, blue; O, red; Zn, light blue; Mo, green; P, yellow. Symmetry codes: #1 *1 – x, – y, z*; #2 *1– x, y, z*; #3 x, – y, z; #4 *1 – x, y, – z*; #5 *x, y,– z*; #6 *1– x, 1 – y, – z*; #7 *x, 1 – y, – z*; #8 *1 – x, 1– y, z*; #9 *x, 1 – y, z*; #10 *1.5 – x, 1 – y, 0.5 – z*; #11 *–0.5 + x, – y, 0.5 – z*. **b** Each Co-TCPP connected four POM fragments. **c** 3D (4,4) connected framework was formed by the 4-connected TCPP linkers and zigzag POM chains. **d** ***mog*** topology. Because M-PMOF crystals are isomorphic, just taking the Co-PMOF for instance here. In this work, we applied polyoxometalates (POMs) and metalloporphyrins to construct polyoxometalate-metalloporphyrin organic frameworks. In the structure, reductive polyoxometalates mainly composed of low valent metal ions, such as Zn-ε-Keggin cluster ({ε-$PMo_8{}^{V}Mo_4{}^{VI}O_{40}Zn_4$}, including eight Mo^{V} atoms), are usually electron-rich aggregates and can easily offer electrons when triggered by redox reaction or bias stimulus. Co-porphyrin, where inherent macrocycle conjugated π-electron system is very beneficial for electron mobility and Co(II) enables to be reduced to Co(I) during the process in many references[1,4-6]. The connection of POM and metalloporphyrin will presumably create an oriented electron transportation pathway under the motivation of electric field, abundant electrons flowing from POM cluster to metalloporphyrin motif can guarantee and facilitate the fulfillment of multiple electron migration process of CO_2RR electrocatalysis.

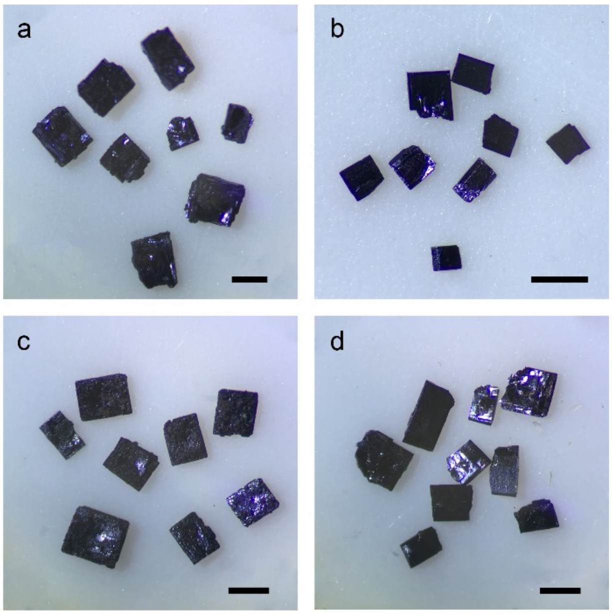

Supplementary Figure 2. Images of M-PMOF. **a** Co-PMOF. **b** Fe-PMOF. **c** Ni-PMOF. **d** Zn-PMOF (scale bar, 500 μm).

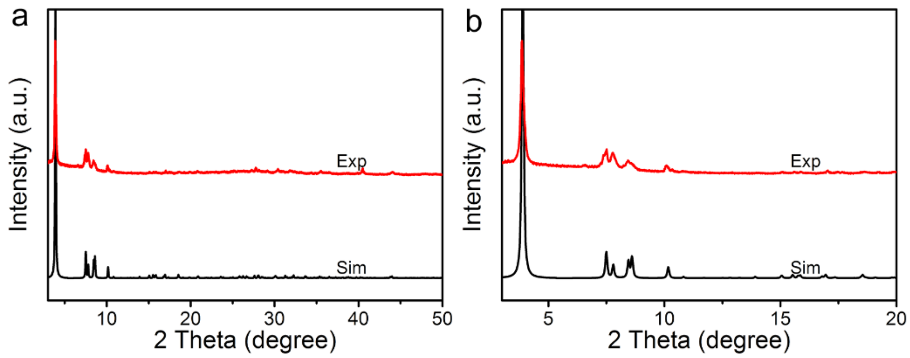

Supplementary Figure 3. PXRD patterns of Co-PMOF. **a** 2 Theta ranges from 3° to 50°. **b** 2 Theta ranges from 3° to 20°. "Sim": simulated pattern and "Exp": as-synthesized sample.

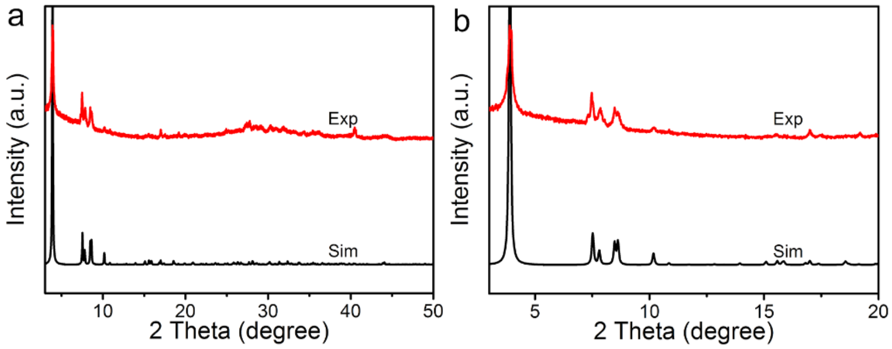

Supplementary Figure 4. PXRD patterns of Fe-PMOF. **a** 2 Theta ranges from 3° to 50°. **b** 2 Theta ranges from 3° to 20°. "Sim": simulated pattern and "Exp": as-synthesized sample.

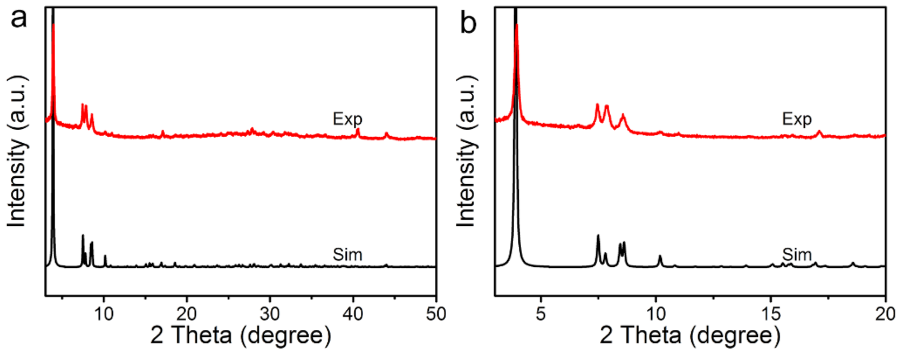

Supplementary Figure 5. PXRD patterns of Ni-PMOF. **a** 2 Theta ranges from 3° to 50°. **b** 2 Theta ranges from 3° to 20°. “Sim”: simulated pattern and “Exp”: as-synthesized sample.

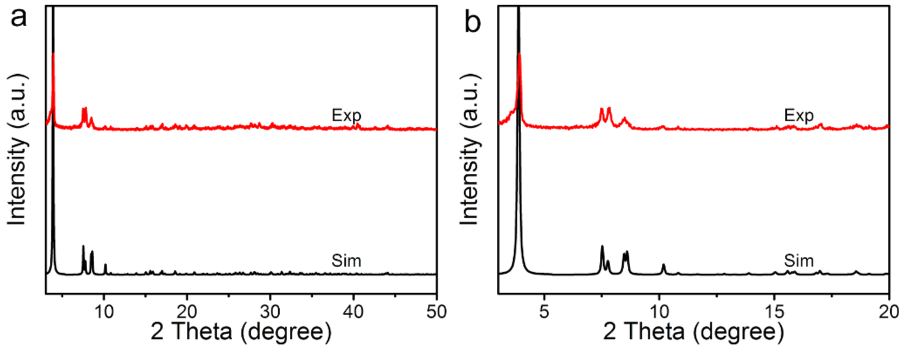

Supplementary Figure 6. PXRD patterns of Zn-PMOF. **a** 2 Theta ranges from 3° to 50°. **b** 2 Theta ranges from 3° to 20°. "Sim": simulated pattern and "Exp": as-synthesized sample.

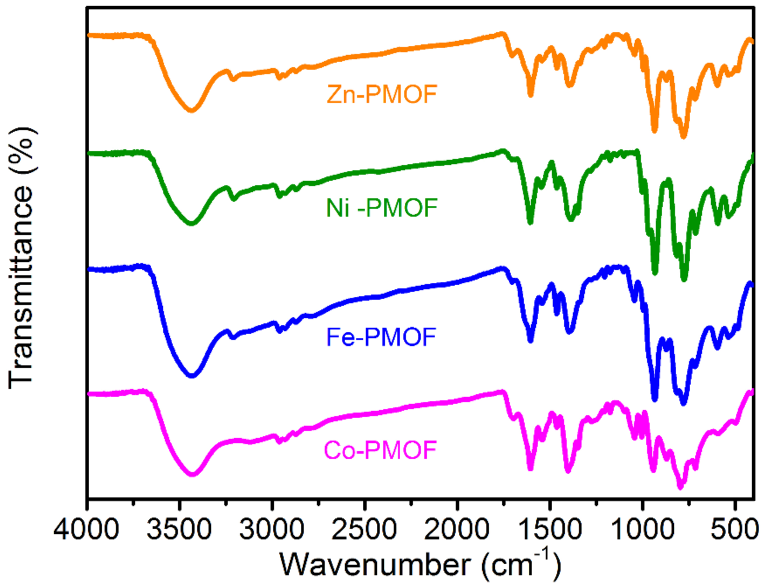

Supplementary Figure 7. IR spectra of Co-PMOF, Fe-PMOF, Ni-PMOF and Zn-PMOF. Taking Co-PMOF for example, the peaks presented can be ascribed to diverse functional groups, they are (υ, cm^{-1}): 1604 (C=C); 1699 (C=O); 1400, 1271 (O-C-O); 1040 (P-O); 1027, 941 (Mo=O); 869, 796 (Mo-O-Mo). IR peak contribution for other PMOFs follows similar principle as Co-PMOF.

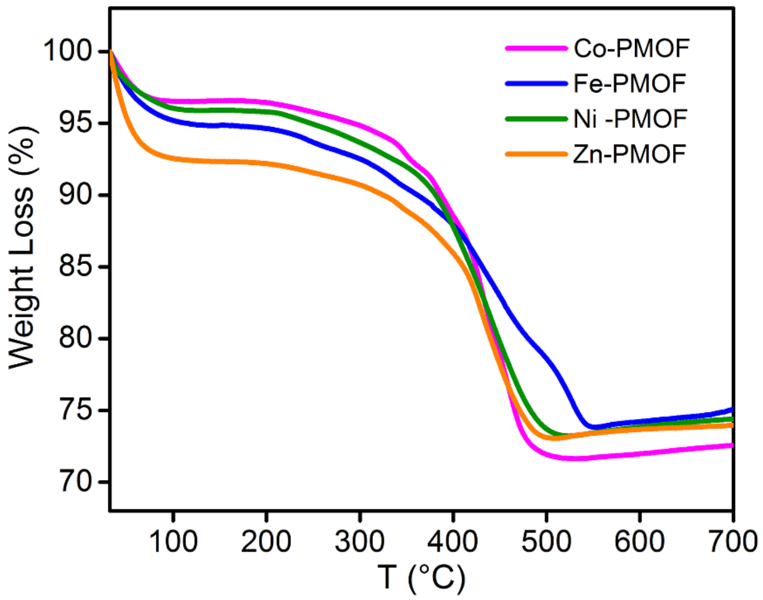

Supplementary Figure 8. Thermogravimetric analyses of Co-PMOF, Fe-PMOF, Ni-PMOF and Zn-PMOF. For the formulae of M-PMOF, all of these M-PMOF are iso-reticular in structure and only have minor difference in centered metal ions. Their structures have been well-defined in the SXRD tests. Based on the elemental analyses and SXRD tests, the calculated formulae was $[PMo^{V}_{8}Mo^{VI}_{4}O_{35}(OH)_{5}Zn_{4}]_{2}$[M-TCPP]$[2H_{2}O]$[1.5TBAOH] (M = Fe, Co, Ni and Zn). This result was further supported by TGA test (Fig. S8). Taking Fe-PMOF for example, in the test, about 6.8% mass loss at temperature range from 0 to 200 °C is attributed to the loss of guest molecules, which matches well with the content of guest molecules in Fe-PMOF. Anal. calcd. (%) for Fe-PMOF: C, 15.95; H, 1.59; N, 1.42. Found: C, 15.65; H, 2.96; N, 1.46.

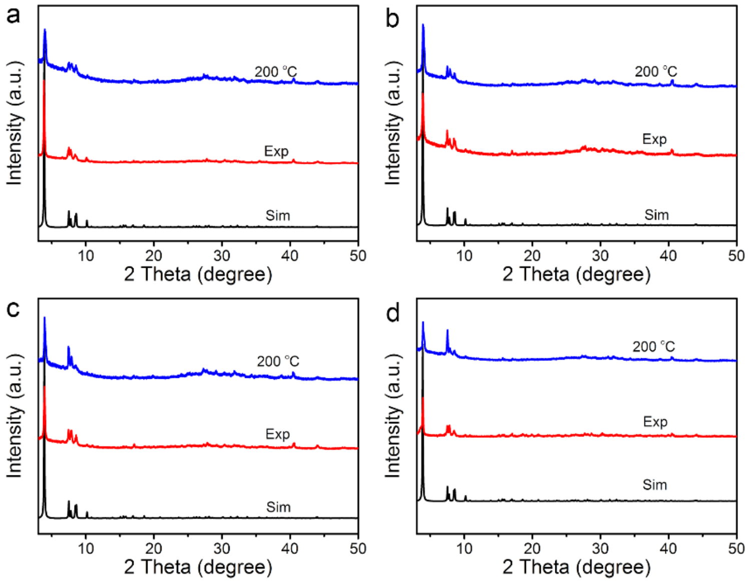

Supplementary Figure 9. PXRD patterns of M-PMOF after thermal treatment. **a** Co-PMOF. **b** Fe-PMOF. **c** Ni-PMOF. **d** Zn-PMOF. All the samples were treated at 200 °C in O_2 atmosphere with a heating rate of 5 °C min^{-1}. "Sim": simulated pattern and "Exp": as-synthesized sample. M-PMOF samples were placed in a tubular furnace and carbonized in the presence of ultrapure O_2 at 200 °C with the heating rate of 5 °C min^{-1}. As shown in the images, the PXRD patterns of M-PMOF still agree well with the simulated ones, indicating the structures of M-PMOF enable to be stable up to 200 °C.

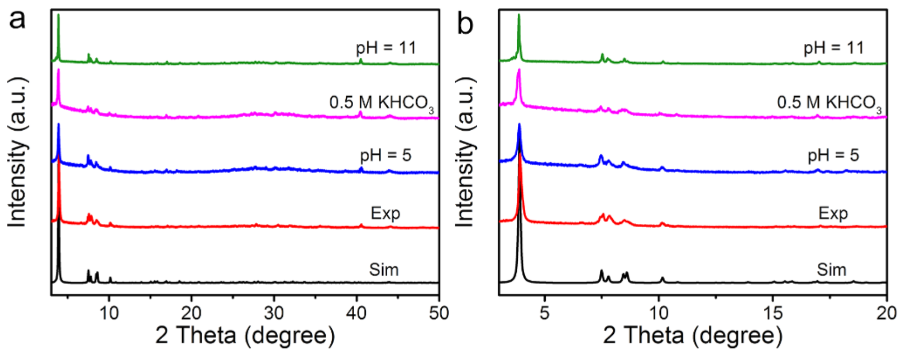

Supplementary Figure 10. PXRD patterns of Co-PMOF in acid, base and 0.5 M $KHCO_3$ solutions. **a** 2 Theta ranges from 3° to 50°. **b** 2 Theta ranges from 3° to 20°. "Sim": simulated pattern and "Exp": as-synthesized sample.

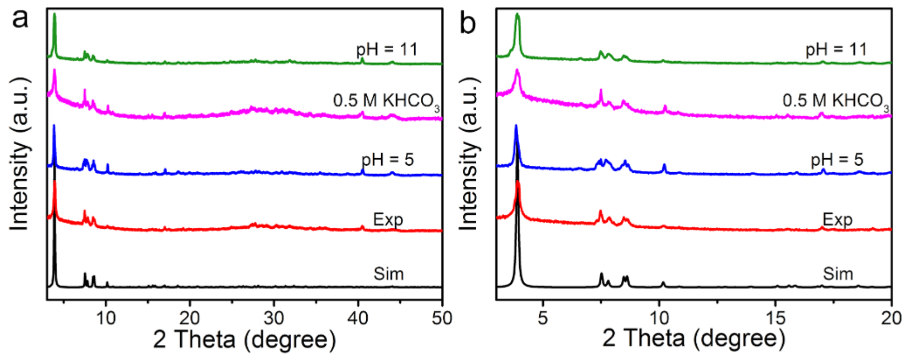

Supplementary Figure 11. PXRD patterns of Fe-PMOF in acid, base and 0.5 M $KHCO_3$ solutions. **a** 2 Theta ranges from 3º to 50º. **b** 2 Theta ranges from 3º to 20º. “Sim”: simulated pattern and “Exp”: as-synthesized sample.

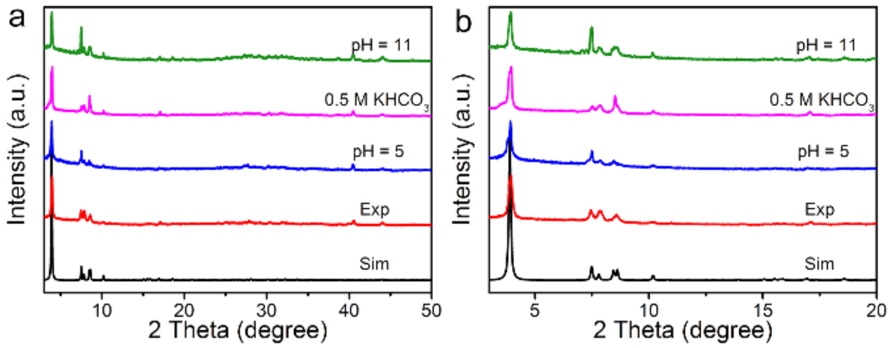

Supplementary Figure 12. PXRD patterns of Ni-PMOF in acid, base and 0.5 M $KHCO_3$ solutions. **a** 2 Theta ranges from 3° to 50°. **b** 2 Theta ranges from 3° to 20°. “Sim”: simulated pattern and “Exp”: as-synthesized sample.

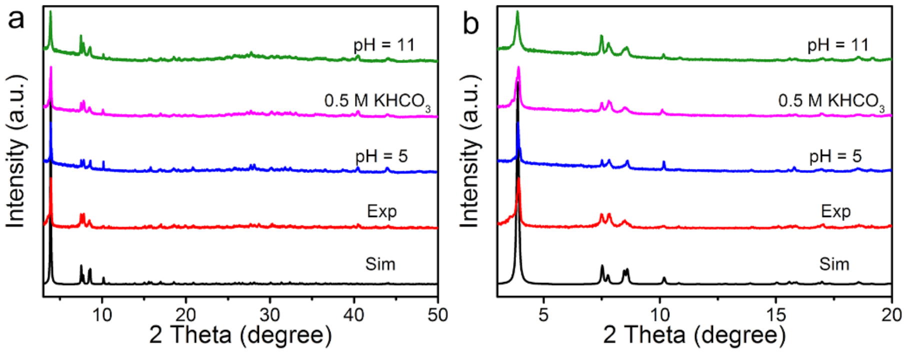

Supplementary Figure 13. PXRD patterns of Zn-PMOF in acid, base and 0.5 M $KHCO_3$ solutions. **a** 2 Theta ranges from 3° to 50°. **b** 2 Theta ranges from 3° to 20°. "Sim": simulated pattern and "Exp": as-synthesized sample.

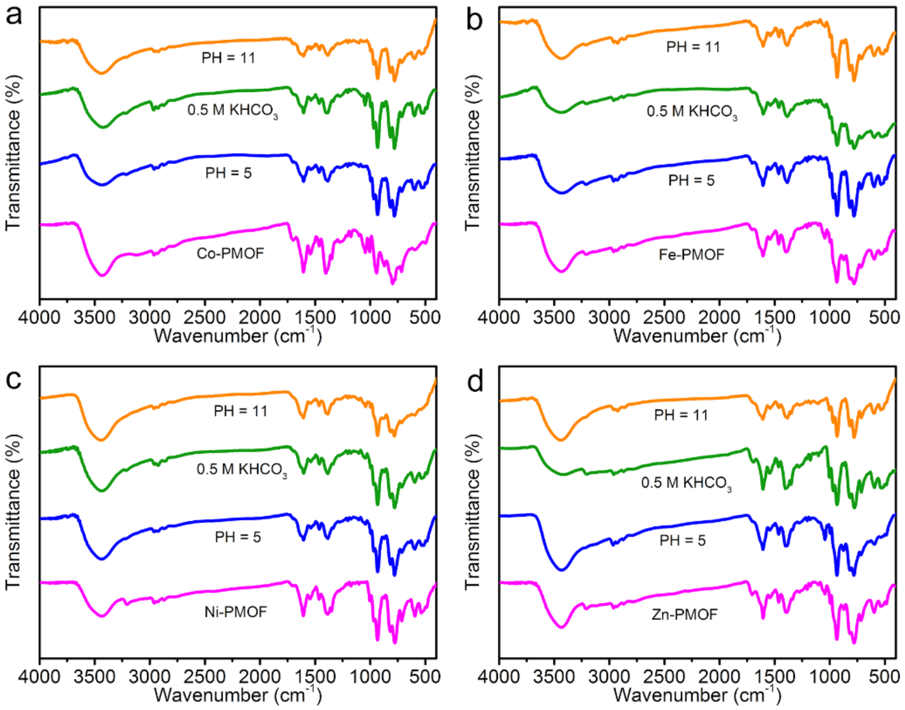

Supplementary Figure 14. IR spectra of M-PMOF in acid, base and 0.5 M $KHCO_3$ solutions at room temperature. **a** Co-PMOF. **b** Fe-PMOF. **c** Ni-PMOF. **d** Zn-PMOF. The IR spectra of M-PMOF in acid, base and 0.5 M $KHCO_3$ solutions have negligible change compared with the as-synthesized ones, indicating M-PMOF can maintain the integrity of their structures after chemical stability tests.

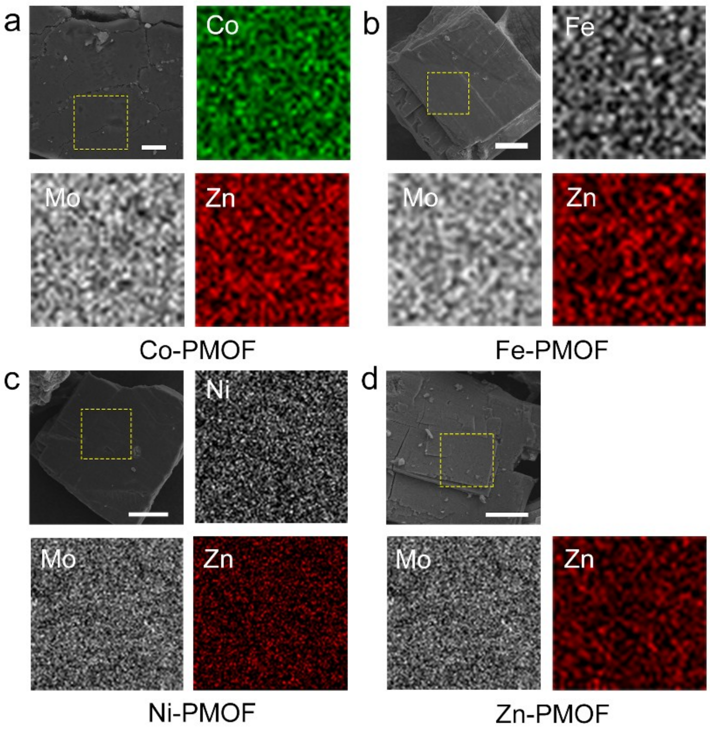

Supplementary Figure 15. SEM image and EDX elemental mapping of M-PNOF after stability test (in acid at room temperature). **a** Co-PMOF. **b** Fe-PMOF. **c** Ni-PMOF. **d** Zn-PMOF (scale bar, 20 μm, 25 μm, 25 μm and 25 μm, respectively). In SEM tests, M-PMOF are all in regular cubic shapes and EDX tests show that metal elements distribute uniformly on the M-PMOF crystals after stability test. Further proved by the ICP leaching test, negligible leaching metal ions were detected in the solution after chemical stability tests.

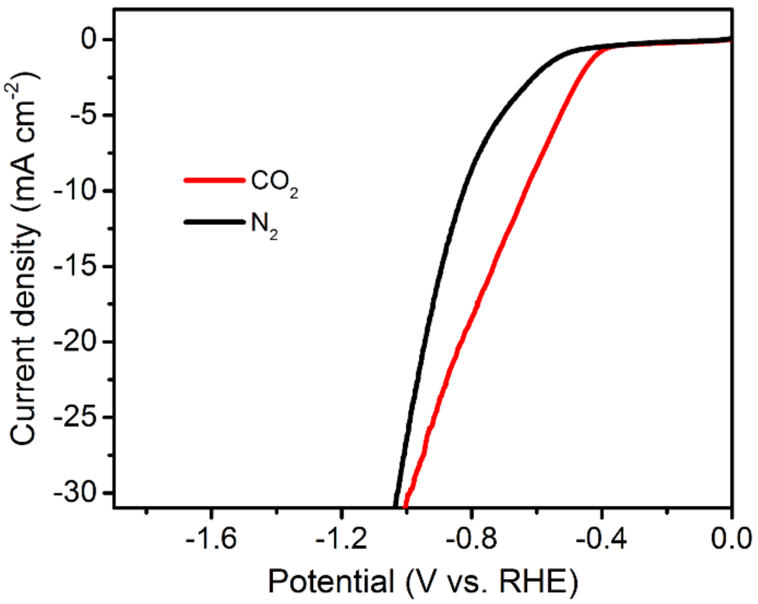

Supplementary Figure 16. Linear sweep voltammetric curves of Co-PMOF in N_2-saturated and CO_2-saturated 0.5 M $KHCO_3$ aqueous solution.

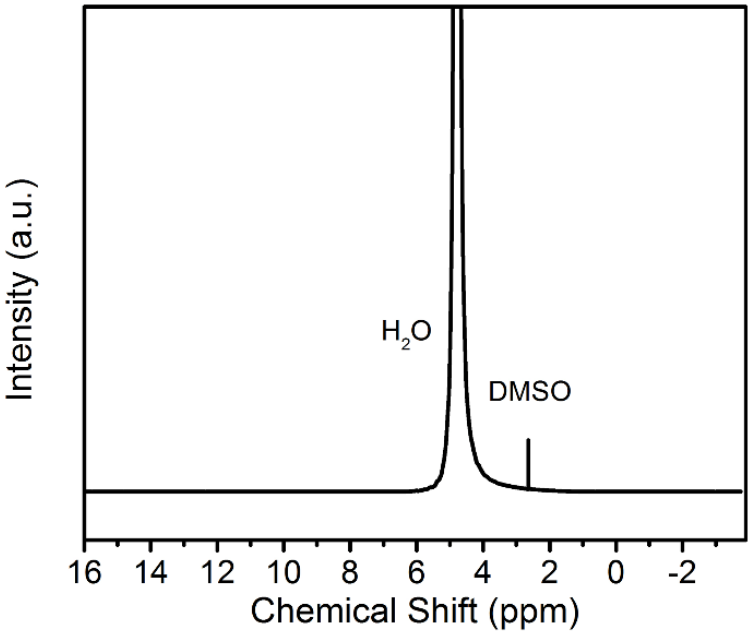

Supplementary Figure 17. Characterization for the liquid product of Co-PMOF during 2-h CO_2 reduction process by ^{1}H nuclear magnetic resonance spectroscopy.

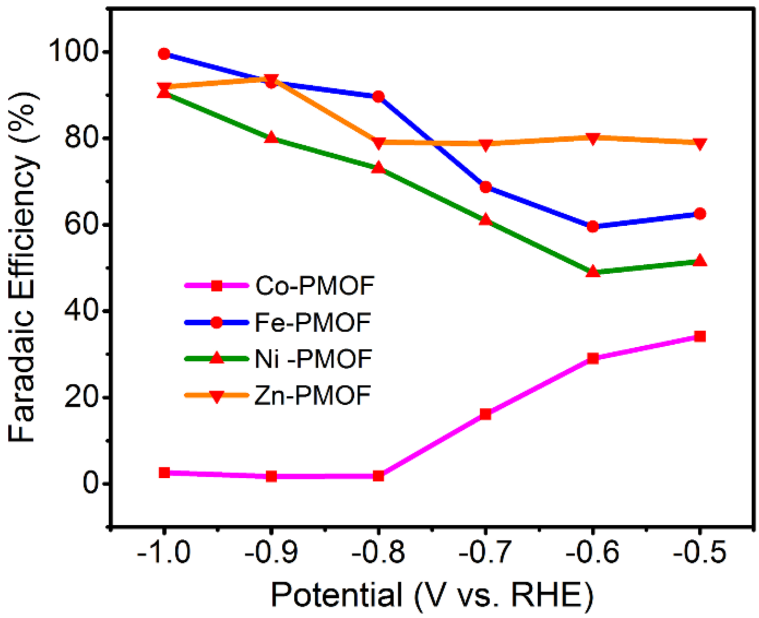

Supplementary Figure 18. Faradaic efficiencies (H_2) of Co-PMOF, Fe-PMOF, Ni-PMOF and Zn-PMOF in CO_2-saturated 0.5 M $KHCO_3$ aqueous solution.

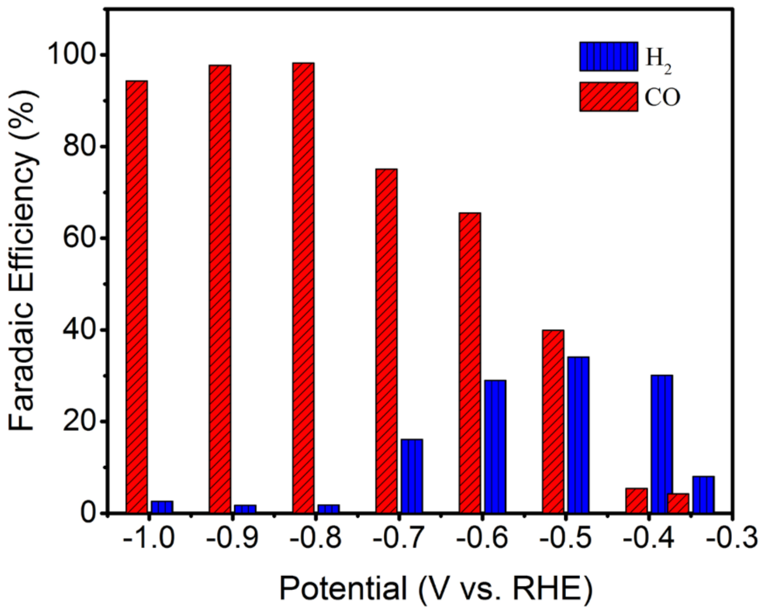

Supplementary Figure 19. Faradaic efficiencies (CO and H_2) of Co-PMOF at different applied potentials in CO_2-saturated 0.5 M $KHCO_3$ aqueous solution.

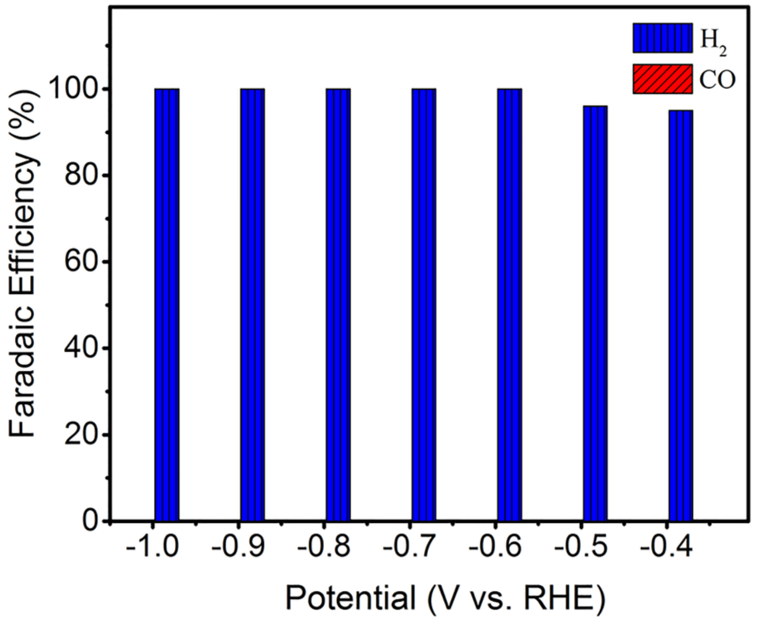

Supplementary Figure 20. Faradaic efficiencies (CO and H_2) of pure carbon cloth at different applied potentials in CO_2-saturated 0.5 M $KHCO_3$ aqueous solution. The bare carbon cloth was measured adopting the same testing methods to conduct its CO_2RR activity. As shown in the image, pure carbon cloth has no CO_2RR activity.

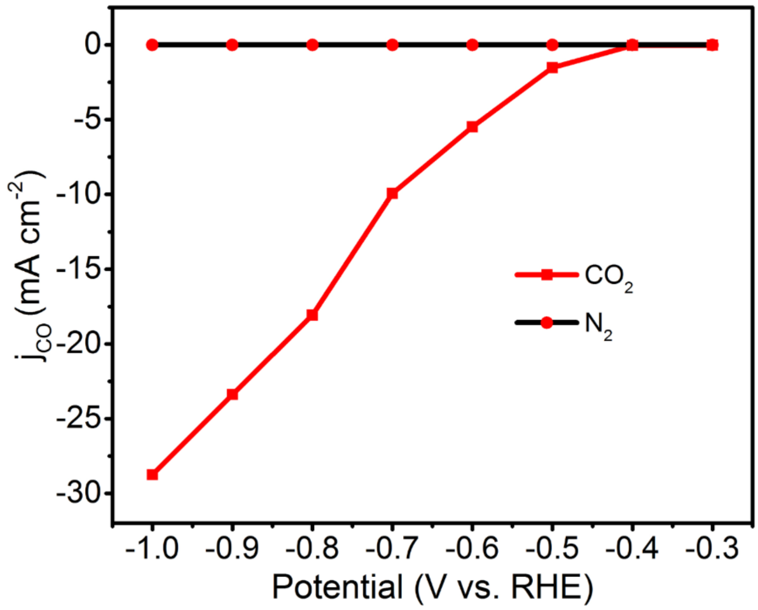

Supplementary Figure 21. CO partial current density of Co-PMOF in N_2-satureted (black) and CO_2-saturated (red) 0.5 M $KHCO_3$ aqueous solution.

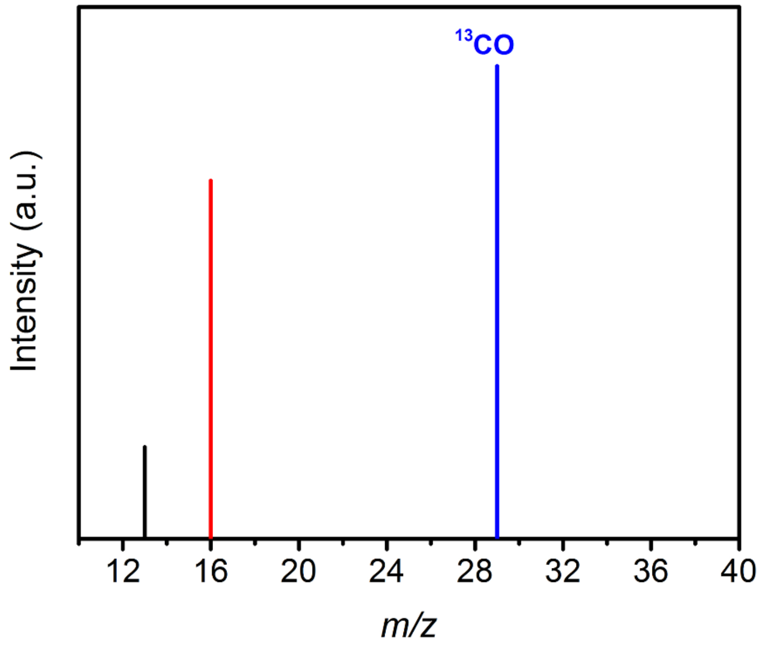

Supplementary Figure 22. The mass spectra of ^{13}CO recorded under $^{13}CO_2$ atmosphere.

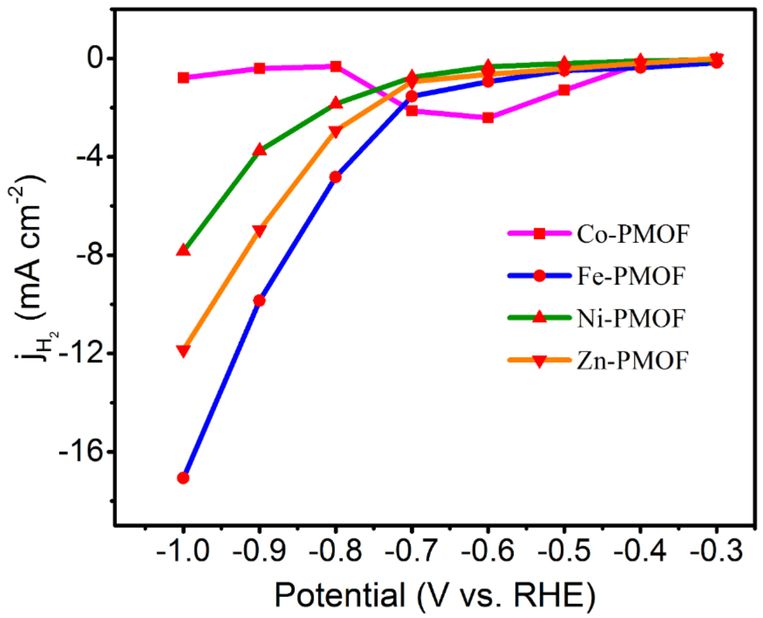

Supplementary Figure 23. Partial H_2 current density (based on geometric surface area) plots of Co-PMOF, Fe-PMOF, Ni-PMOF and Zn-PMOF in CO_2-saturated 0.5 M $KHCO_3$ aqueous solution.

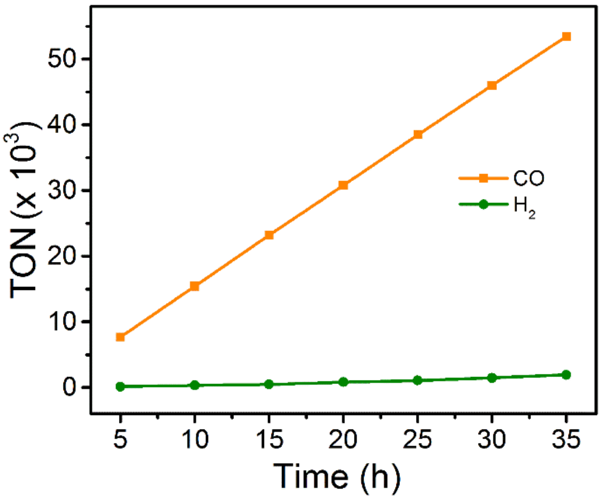

Supplementary Figure 24. Plots of CO and H_2 evolving turnover number versus time for Co-PMOF. As shown in the images, the TON (H_2) is close to 0 owing to its low efficiency for H_2 generation. Notably, the TON (CO) is as high as 7693 in just 5 h and can reach up to 53433 after 35 h.

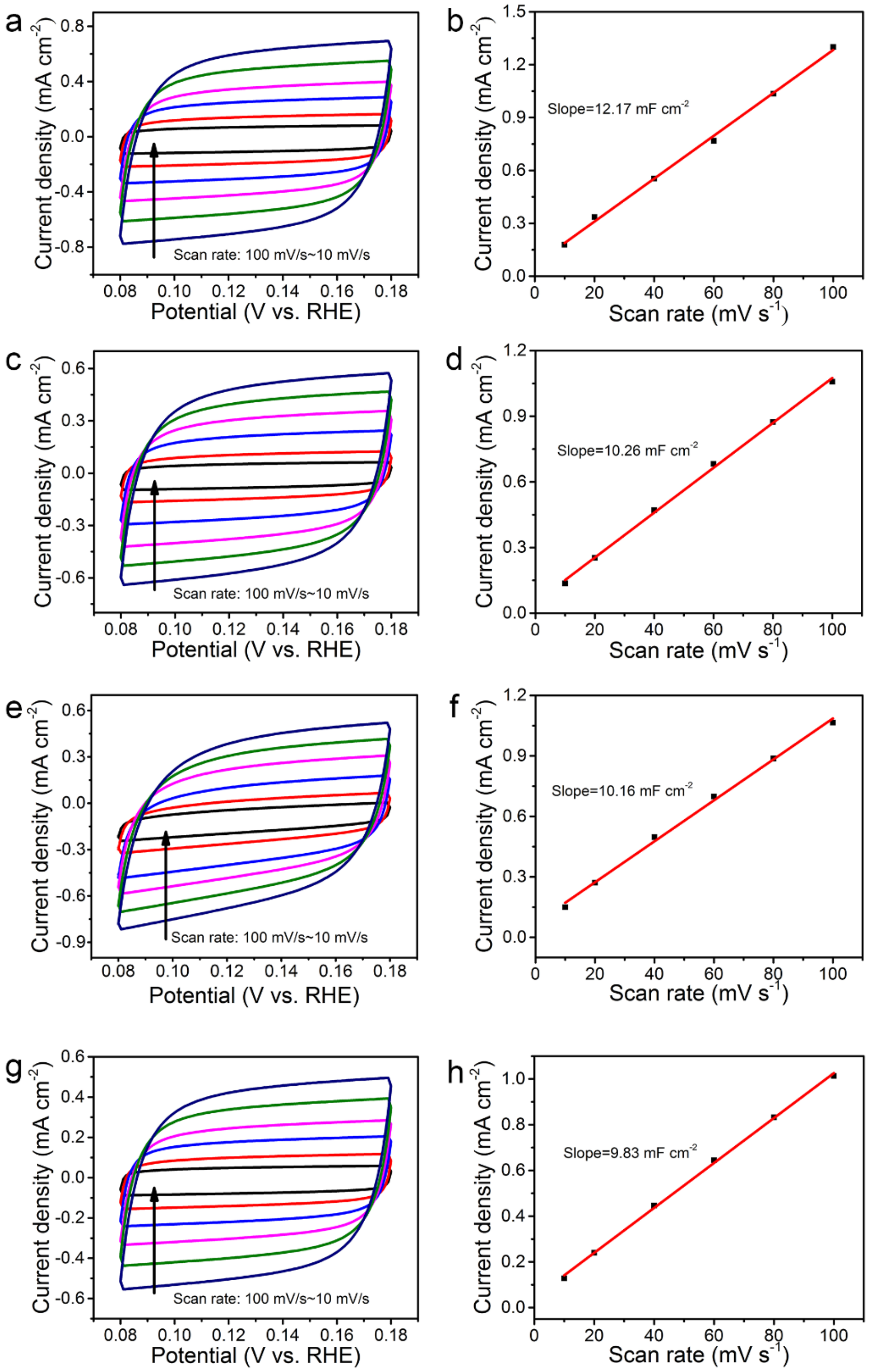

Supplementary Figure 25. Cyclic voltammertrys (CV) curves in the region of 0.08 ~ 0.18 V *vs*. RHE at various scan rate (10 ~ 100 mV s^{-1}) and corresponding capacitive current at 0.13 V as a function of scan rate for **a, b** Co-PMOF. **c, d** Fe-PMOF. **e, f** Ni-PMOF. **g, h** Zn-PMOF.

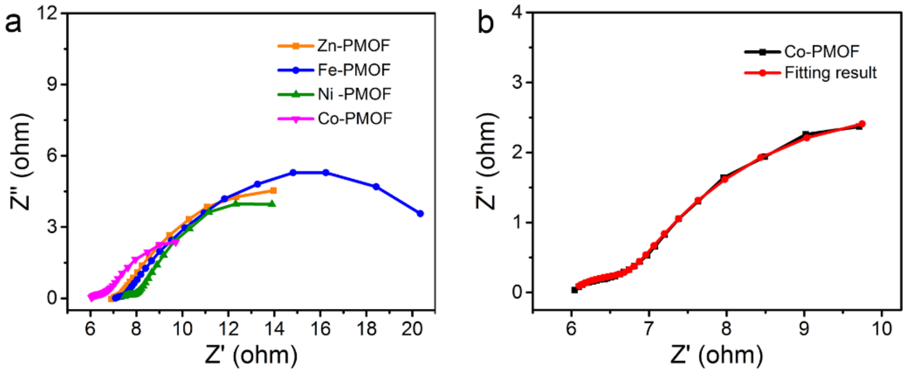

Supplementary Figure 26. Nyquist plots of electrocatalysts over the frequency ranging from 1000 kHz to 0.1 Hz at -0.83 V *vs*. RHE.

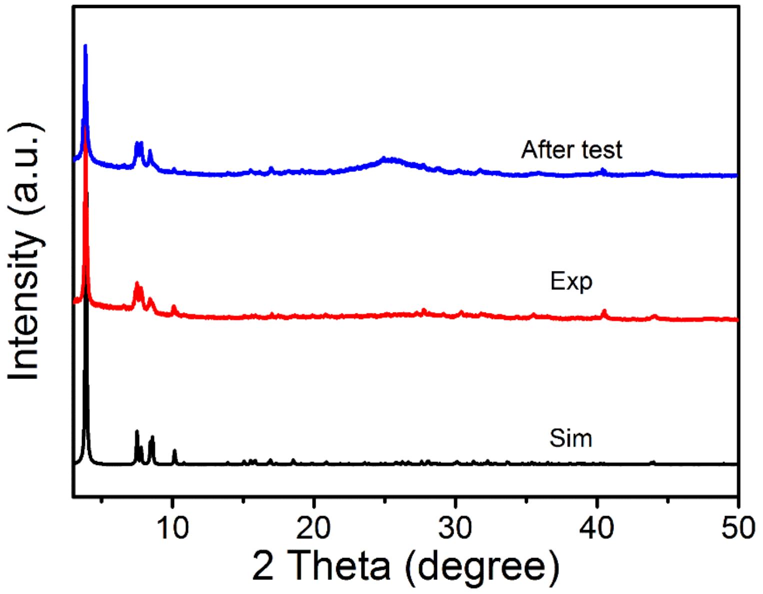

Supplementary Figure 27. PXRD patterns of Co-PMOF after electrochemical experiment. “Sim”: simulated pattern and “Exp”: as-synthesized sample. The PXRD pattern of Co-PMOF after electrochemical experiment matches well with the simulated and as-synthesized samples.

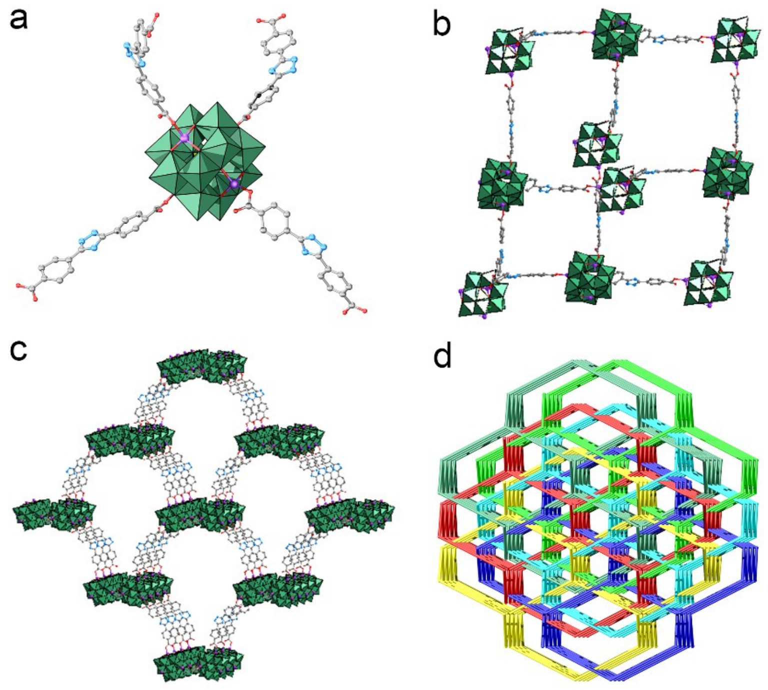

Supplementary Figure 28. The structure images of NNU-12. **a** Secondary building block. **b** Basic construction unit. **c** 3D framework. **d** Six-fold interpenetrated structure with a ***dia*** topology. As presented in the image, each $BCPT^{2-}$ ligand connects two Zn-ε-Keggin segments and each Zn-ε-Keggin connects four ligands, which generates a 3D framework with six-fold interpenetrated structure with a ***dia*** topology. NNU-12 contains the same Zn-ε-Keggin unit as Co-PMOF while the ligand is different.

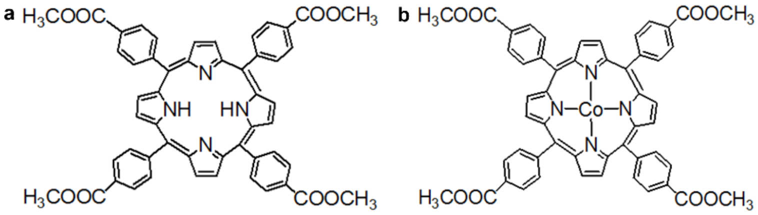

Supplementary Figure 29. The structure images of TMCP and Co-TMCP. **a** TMCP. **b** Co-TMCP. TMCP is a kind of ester compound and Co-TMCP is a kind of Co-centered macrocycle.

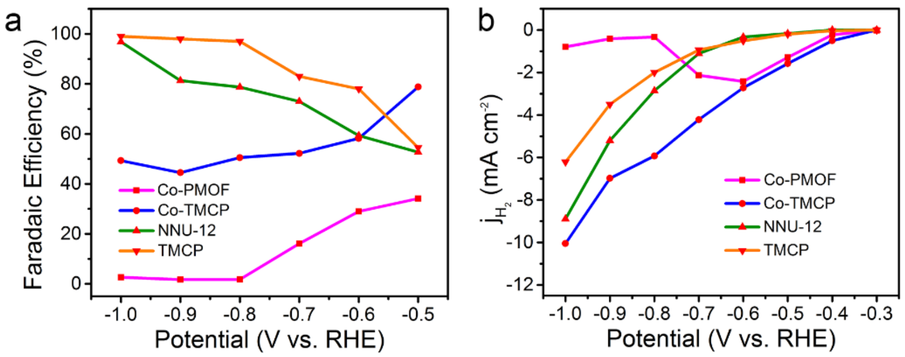

Supplementary Figure 30. a Faradaic efficiencies and **b** partial current density (based on geometric surface area) plots for H_2 of Co-PMOF, Co-TMCP, NNU-12 and TMCP in CO_2-saturated 0.5 M $KHCO_3$ aqueous solution.

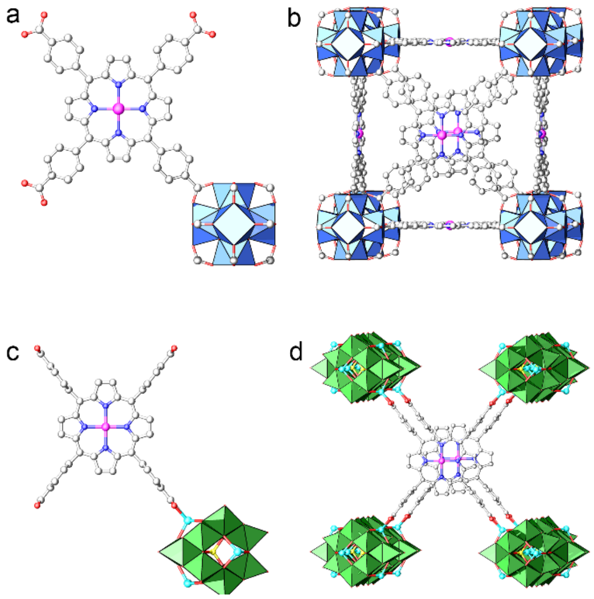

Supplementary Figure 31. a The coordination environment of $Zr_6O_4(OH)_4$ unit in MOF-525(Co). **b** MOF-525(Co). **c** The coordination environment of Zn-ε-Keggin unit in Co-PMOF. **d** Co-PMOF. Co-PMOF was constructed by the 4-connected TCPP linkers and zigzag POM chains. MOF-525(Co) has similar ligand as Co-PMOF and is constructed with $Zr_6O_4(OH)_4$ unit without POM. In the structure of MOF-525(Co), each $Zr_6O_4(OH)_4$ unit connects with twelve $TCPP^{4-}$ ligand to generate a 3D framework with a ***ftw*** topology[18].

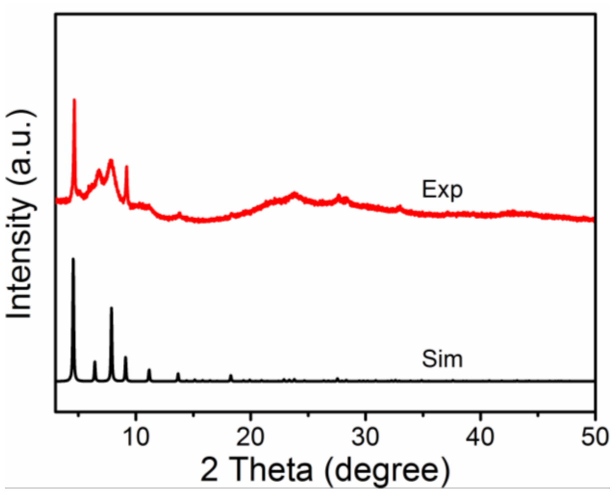

Supplementary Figure 32. PXRD patterns of MOF-525(Co)[18]. “Sim”: simulated pattern and “Exp”: as-synthesized sample.

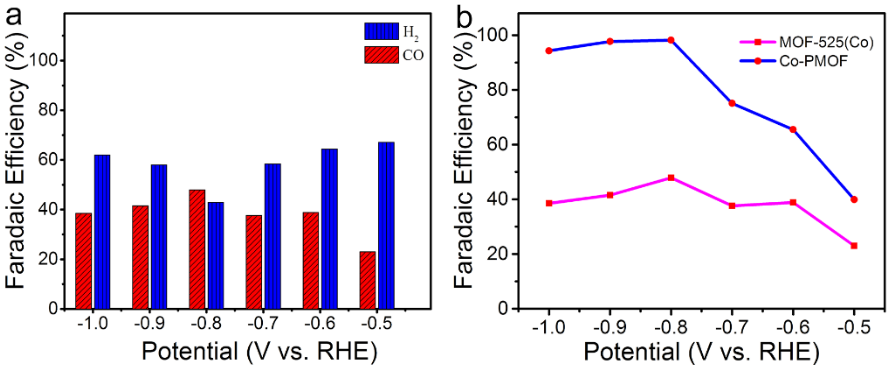

Supplementary Figure 33. Faradaic efficiencies of MOF-525(Co) and Co-PMOF at different applied potentials in CO_2-saturated 0.5 M $KHCO_3$ aqueous solution. **a** Faradaic efficiencies (CO and H_2) of MOF-525(Co). **b** Faradaic efficiencies (CO) of MOF-525(Co) and Co-PMOF.

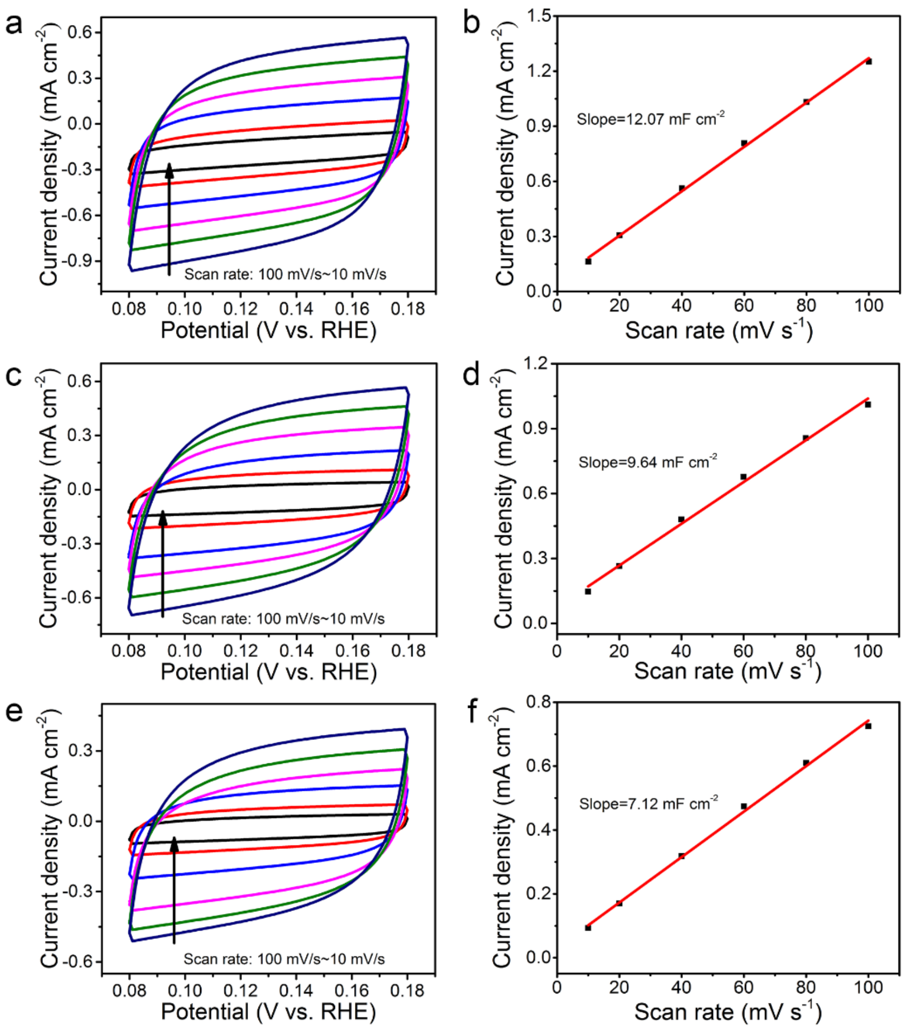

Supplementary Figure 34. Cyclic voltammertrys (CV) curves in the region of 0.08 ~ 0.18 V *vs*. RHE at various scan rate (10 ~ 100 mV s^{-1}) and corresponding capacitive current at 0.13 V as a function of scan rate for **a, b** Co-TMCP. **c, d** NNU-12. **e, f** TMCP.

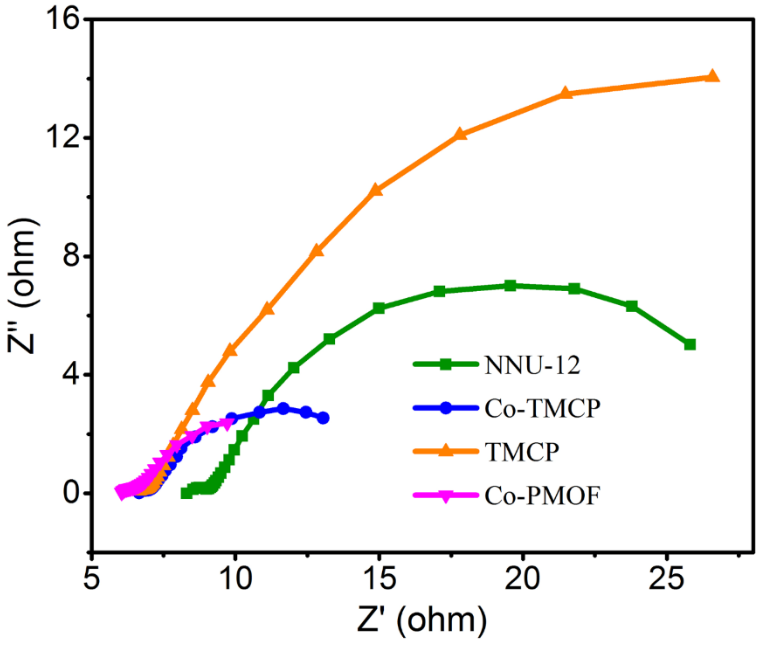

Supplementary Figure 35. Nyquist plots of electrocatalysts over the frequency ranging from 1000 kHz to 0.1 Hz at -0.83 V *vs*. RHE.

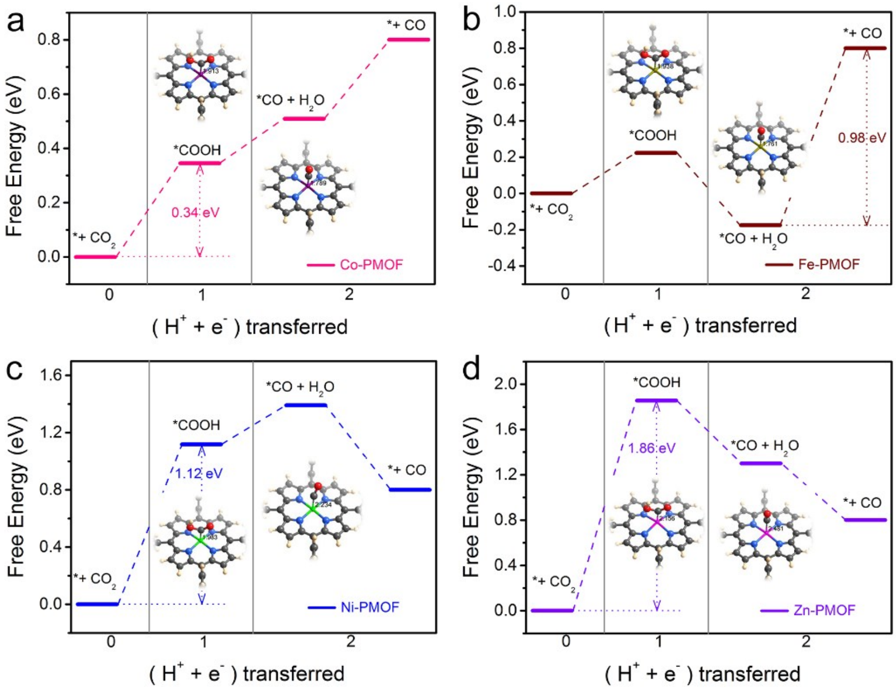

Supplementary Figure 36. The free energy diagrams of CO_2 reduction to CO for M-PMOF. **a** Co-PMOF. **b** Fe-PMOF. **c** Ni-PMOF. **d** Zn-PMOF. The corresponding configurations of the adsorbed intermediates of *COOH and *CO are shown.

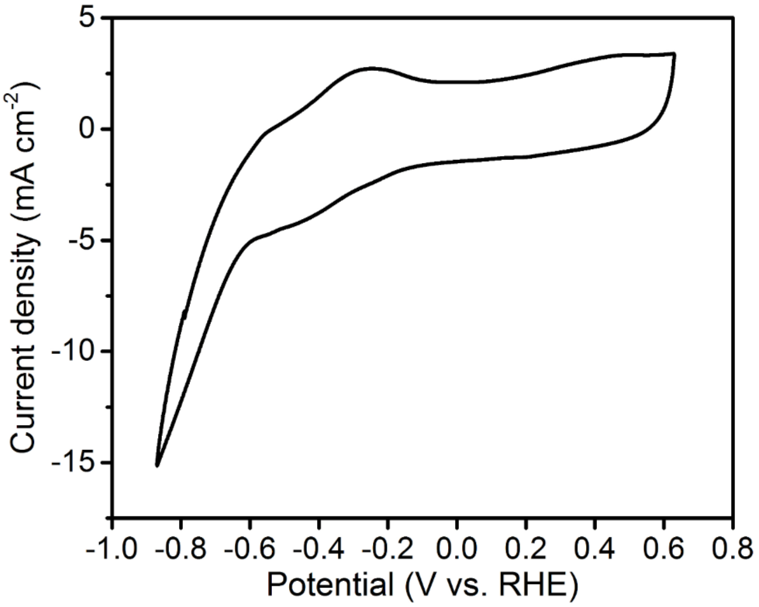

Supplementary Figure 37. CV curve of Co-PMOF in N_2-saturated 0.5 M $KHCO_3$.

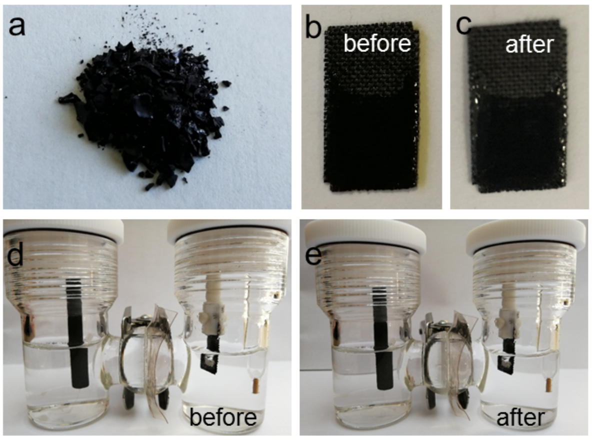

Supplementary Figure 38. The photo images of Co-TMCP before and after CO_2RR test. **a** Co-TMCP powder. **b** Co-TMCP loaded carbon cloth electrode before CO_2RR test. **c** Co-TMCP loaded carbon cloth electrode after CO_2RR test. **d** The electrolyte before CO_2RR test. **e** The electrolyte after CO_2RR test. After electrocatalysis, the solution has no color change.

Supplementary Table 1. CO_2 electroreduction performances of Co-PMOF and other electrocatalysts.

Catalysts	Electrolyte & pH	E (V *vs.* SHE)	Main product	FE (%)	TOF (h^{-1})	Ref.
Co-PMOF	0.5 M $KHCO_3$ pH = 7.2	-0.8 V (*vs.* RHE)	CO	98.7	1656	This work
Fe-PMOF	0.5 M $KHCO_3$ pH = 7.2	-0.7 V (*vs.* RHE)	CO	28.8	17.45	This work
Ni-PMOF	0.5 M $KHCO_3$ pH = 7.2	-0.8 V (*vs.* RHE)	CO	18.5	8.11	This work
Zn-PMOF	0.5 M $KHCO_3$ pH = 7.2	-0.9 V (*vs.* RHE)	CO	0.95	0.005	This work
CR-MOF	0.5 M $KHCO_3$ pH = 7.2	-1.2 V	HCOOH	30	NA[a]	7
HKUST-1	0.01 M $tbaBF_4$/DMF	-2.2 V	Oxalic acid	51	NA	8
CuAdeAce	0.5 M KCO_3	-1.55 V	MeOH	1.2	0.002	9
HKUST-1	0.5 M KCO_3	-0.7 V	EtOH	15.9	0.02	9
$[Al_2(OH)_2(Co(tcpp))]$	0.5 M KCO_3	-0.7 V (*vs.* RHE)	CO	76	200	4
Fe-MOF-525	1 M $tbaPF_6$ in CH_3CN	-1.3 V	CO	60	469	10
Re-SURMOF	0.1 M tbaOH in CH_3CN	-0.9 V	CO	92.9	690	11
Cu-SIM NU-1000	0.1 M $NaClO_4$	-0.82 V (*vs.* RHE)	$HCOO^-$	31	NA	12
COF-367-Co	potassium phosphate buffer PH = 7.2	-0.67 V (*vs.* RHE)	CO	90	9400	3
Ni SAs/N-C	0.5 M $KHCO_3$	-1 V (*vs.* RHE)	CO	70.3	5273	13

Nanoporous Ag	0.5 M $KHCO_3$	-0.5 V (*vs.* RHE)	CO	92	7.2	14
Pd Nanoparticles	0.1 M $KHCO_3$	-0.89 V (*vs.* RHE)	CO	91	576	15
N-based silver catalyst	1 M KOH	-1.6 V	CO	90	2000	16
CATPyr/CNT	0.5 M $KHCO_3$	-0.59 V (*vs.* RHE)	CO	93	144	17

Note: [a] NA means not mentioned in article.

As presented in the Supplementary Table 1, the maximum FE_{CO} value (98.7%) of Co-PMOF is higher than other M-PMOFs (i.e. Fe-PMOF, 28.8%; Ni-PMOF, 18.5% and Zn-PMOF, 1.2%, respectively) and is highest among reported MOFs. Besides, TOF of Co-PMOF is calculated to be 1656 h^{-1} at -0.8 V. To the best of our knowledge, this catalytic behavior of Co-PMOF outperforms most of MOF catalysts and is one of the best reported materials.

Supplementary Table 2. Crystal data and structure refinement for **M-PMOFs**.

	**Co-PMOF**	**Fe-PMOF**	**Ni-PMOF**	**Zn-PMOF**
**Empirical formulae**	$C_{48}H_{26}CoMo_{24}N_4O_{89}P_2Zn_8$	$C_{48}H_{26}FeMo_{24}N_4O_{89}P_2Zn_8$	$C_{48}H_{24}Mo_{24}N_4NiO_{88}P_2Zn_8$	$C_{12}H_{6.50}Mo_6NO_{22.25}P_{0.50}Zn_{2.25}$
**Formulae weight**	5029.12	5026.04	5010.88	1258.93
**Crystal system**	Orthorhombic	Orthorhombic	Orthorhombic	Orthorhombic
**Space group**	*Fmmm*	*Fmmm*	*Fmmm*	*Fmmm*
a **(Å)**	17.386(4)	17.3721(12)	17.352(5)	23.478(2)
b **(Å)**	23.565(6)	23.4822(17)	23.578(7)	45.478(5)
**c (Å)**	45.325(11)	45.243(3)	45.260(13)	17.3475(19)
α **(°)**	90	90	90	90
β **(°)**	90	90	90	90
γ **(°)**	90	90	90	90
V **($Å^3$)**	18569(8)	18456(2)	18518(9)	18522(3)
Z	4	4	4	16
**D_{calc}($Mg·m^{-3}$)**	1.799	1.809	1.797	1.806
**Abs.coeff.(mm^{-1})**	2.741	2.747	2.760	2.788
F**(000)**	9436.0	9432.0	9400.0	9443.0
**Reflns collected**	65134	30965	29915	24124
**Independent reflns**	5702	5674	3893	5368
**GOFon F^2**	1.120	1.108	1.146	1.159
**R_{int}**	0.065	0.0269	0.0599	0.0262
**R_1 [$I > 2\sigma(I)$][a]**	0.0495	0.0281	0.1238	0.0394
**wR_2 [$I > 2\sigma(I)$][a]**	0.1228	0.0800	0.2686	0.0928
**R_1(all data)[b]**	0.0677	0.0337	0.1517	0.0505
**wR_2(all data)[b]**	0.1398	0.0838	0.2888	0.1009

[a] $R_1 = \Sigma||F_o| - |F_c||/\Sigma|F_o|$. [b] $wR_2 = |\Sigma w(|F_o|^2 - |F_c|^2)|/\Sigma|w(F_o^2)^2|^{1/2}$.

Supplementary Note 1: Reaction product analysis and calculation.

The bulk electrolysis was carried out in an airtight electrochemical H-type cell at selected potentials (-0.5 to -1 V) to determine the reduction products and their Faradic efficiency. The gaseous reduction products (e.g., CO) were monitored by a gas chromatography (GC-7920) equipped with a flame ionization detector (FID). During the test, helium was the carrier gas. A thermal conductivity detector (TCD) was used to analyze hydrogen with nitrogen as the carrier gas.

The liquid products were collected from the cathode chambers after electrolysis and quantified by NMR (Bruker AVANCEAV III 400) spectroscopy, in which 0.5 mL electrolyte was mixed with 0.1 mL D_2O and 0.1 µL dimethyl sulfoxide (DMSO, 99.99%, internal standard). Solvent pre-saturation technique was implemented to suppress the water peak.

The calculation of Faradaic efficiency

For CO,

$$\text{FE}=\frac{2F\times n_{co}}{I\times t}\times 100\% \quad \text{(Supplementary Equation 1)}$$

For H_2,

$$\text{FE}=\frac{2F\times n_{H_2}}{I\times t}\times 100\% \quad \text{(Supplementary Equation 2)}$$

where F is the Faraday constant, n_{co} is the moles of produced CO and n_{H_2} is the moles of produced H_2.

Turnover Frequency (TOF, h^{-1})

The TOF for CO was calculated as follow:

$$\text{TOF} = \frac{I_{product}/NF}{m_{cat}\times\omega/M_{Co}}\times 3600 \quad \text{(Supplementary Equation 3)}$$

$I_{product}$: partial current for certain product, CO;

N: the number of electron transferred for product formation, which is 2 for CO;

F: Faradaic constant, 96485 C mol^{-1};

m_{cat}: catalyst mass in the electrode, g;

ω: Co loading in the catalyst;

M_{Co}: atomic mass of Co, 58.93 g mol^{-1}.

Turnover number (TON) is defined as the mole of reduction product generated per electrocatalytic active site over a given period of time.

The TON for CO was calculated as follows[1]:

$$\text{TON}=\frac{Q\times FE_{co(average)}}{2F\times n_{tot}} \quad \text{(Supplementary Equation 4)}$$

Supplementary Note 2: DFT calculations.

Electronic calculations were performed by the spin polarization density functional theory (DFT) in the Dmol^3 module of Materials Studio 5.5 package. The generalized gradient approximation (GGA) with Perdew-Becke-Ernzerhof (PBE) was used for the exchange-correlation function. The double numerical plus d-functions (DND) basis set was adopted, while an accurate DFT Semi-core Pseudopots (DSPP) was employed for the metal atoms. The thermal smearing was applied to the orbital occupation to speed up convergence. For all the DFT calculations, the energy, gradient and displacement convergence criterions were set as 1×10^{-5} Ha, 2×10^{-3} Å and 5×10^{-3} Å, respectively. The ground state structures of *COOH and *CO adsorbed on the catalyst surfaces were determined by searching all the possible configurations on possible active sites till found that of lowest energy without considering the solvation effects. The free energy of the adsorbed state was calculated as:

$$\Delta G = \Delta E + \Delta E_{\mathrm{ZPE}} - T\Delta S + \int \mathrm{CpdT}$$

where T is the temperature, E is the electronic energy calculated by DFT; E_{ZPE} and S are the zero point energy and entropy, respectively, estimated under harmonic approximation from the frequency analysis. The ∫CpdT is small for the adsorbates compared to E and E_{ZPE}, which can be neglected in this study[2, 3].

Supplementary Note 3: The possible catalytic mechanism.

In N_2-saturated 0.5 M $KHCO_3$:

Co(II)PMOF + e → Co(I)PMOF$^-$

Co(I)PMOF$^-$ - e → Co(II)PMOF

In CO_2-saturated 0.5 M $KHCO_3$:

Co(II)PMOF + e → Co(I)PMOF$^-$

Co(I)PMOF$^-$ + CO_2 → $^*CO_2$Co(II)PMOF$^-$

$^*CO_2$Co(II)PMOF$^-$ + H^+ + e → $^*CO_2$HCo(II)PMOF$^-$

$^*CO_2$HCo(II)PMOF$^-$ + H^+ → Co(II)PMOF + CO + H_2O

Supplementary References

1. Han, N. et al. Supported cobalt polyphthalocyanine for high-performance electrocatalytic CO_2 reduction. *Chem* **3**, 652-664 (2017).
2. Peterson, A. A. How copper catalyzes the electroreduction of carbon dioxide into hydrocarbon fuels. *Energy Environ. Sci.* **3**, 1311-1315 (2010).
3. Li, X. et al. Exclusive Ni-N_4 sites realize near-unity CO selectivity for electrochemical CO_2 reduction. *J. Am. Chem. Soc.* **139**, 14889-14892 (2017).
4. Lin, S. et al. Covalent organic frameworks comprising cobalt porphyrins for catalytic CO_2 reduction in water. *Science* **349**, 1208-1213 (2015).
5. Kornienko, N. et al. Metal-organic frameworks for electrocatalytic reduction of carbon dioxide. *J. Am. Chem. Soc.* **137**, 14129-14135 (2015).
6. Sende, J. A. R. et al. Electrocatalysis of CO_2 reduction in aqueous media at electrodes modified with electropolymerized films of vinylterpyridine complexes of transition metals. *Inorg. Chem.* **34**, 3339-3348 (1995).
7. Hinogami, R. et al. Electrochemical reduction of carbon dioxide using a copper rubeanate metal organic framework. *ECS Electrochem. Lett.* **1**, H17-H19 (2012).
8. Kumar, R. S., Kumar, S. S. & Kulandainathan, M. A. Highly selective electrochemical reduction of carbon dioxide using Cu based metal organic framework as an electrocatalyst. *Electrochem. Commun.* **25**, 70-73 (2012).
9. Albo, J. et al. Copper-based metal-organic porous materials for CO_2 electrocatalytic reduction to alcohols. *ChemSusChem* **10**, 1-11 (2016).
10. Hod, I. et al. Fe-porphyrin-based metal-organic framework films as high-surface concentration, heterogeneous catalysts for electrochemical reduction of CO_2. *ACS Catal.* **5**, 6302-6309 (2015).
11. Ye, L. et al. Highly oriented MOF thin film-based electrocatalytic device for the reduction of CO_2 to CO exhibiting high faradic efficiency. *J. Mater. Chem. A* **4**, 15320-15326 (2016).
12. Kung, C. W. et al. Copper nanoparticles installed in metal-organic framework thin films are electrocatalytically competent for CO_2 reduction. *ACS Energy Lett.* **2**, 2394-2401 (2017).
13. Zhao, C. et al. Ionic exchange of metal-organic frameworks to access single nickel sites for efficient electroreduction of CO_2. *J. Am. Chem. Soc.* **139**, 8078-8081 (2017).
14. Lu, Q. et al. A selective and efficient electrocatalyst for carbon dioxide reduction. *Nat. Commun.* **5**, 3242 (2014).
15. Gao, D. et al. Size-dependent electrocatalytic reduction of CO_2 over Pd nanoparticles. *J. Am. Chem. Soc.* **137**, 4288-4291 (2015).
16. Tornow, C. E. et al. Nitrogen-based catalysts for the electrochemical reduction of CO_2 to CO. *J. Am. Chem. Soc.* **134**, 19520-19523 (2012).
17. Maurin, A. & Robert, M. Noncovalent immobilization of a molecular iron-based electrocatalyst on carbon electrodes for selective, efficient CO_2-to-CO conversion in water. *J. Am. Chem. Soc.* **138**, 2492-2495 (2016).
18. Morris, W. et al. Synthesis, structure, and metalation of two new highly porous zirconium metal–organic frameworks. *Inorg. Chem.* **51**, 6443-6445 (2012).
